# Resolving fine‐scale population structure and fishery exploitation using sequenced microsatellites in a northern fish

**DOI:** 10.1111/eva.12922

**Published:** 2020-02-20

**Authors:** Kara K. S. Layton, Brian Dempson, Paul V. R. Snelgrove, Steven J. Duffy, Amber M. Messmer, Ian G. Paterson, Nicholas W. Jeffery, Tony Kess, John B. Horne, Sarah J. Salisbury, Daniel E. Ruzzante, Paul Bentzen, David Côté, Cameron M. Nugent, Moira M. Ferguson, Jong S. Leong, Ben F. Koop, Ian R. Bradbury

**Affiliations:** ^1^ Department of Ocean Sciences Memorial University of Newfoundland St. John's NL Canada; ^2^ Fisheries and Oceans Canada Northwest Atlantic Fisheries Centre St. John's NL Canada; ^3^ Department of Biology Dalhousie University Halifax NS Canada; ^4^ Fisheries and Oceans Canada Bedford Institute of Oceanography Dartmouth NS Canada; ^5^ National Oceanic and Atmospheric Administration Southwest Fisheries Science Center La Jolla CA USA; ^6^ Department of Integrative Biology University of Guelph Guelph ON Canada; ^7^ Department of Biology University of Victoria Victoria BC Canada; ^8^ Centre for Biomedical Research University of Victoria Victoria BC Canada

**Keywords:** genetic assignment, genome‐wide polymorphisms, mixed stock analysis, *Salvelinus alpinus*, sequenced microsatellites, tagging

## Abstract

The resiliency of populations and species to environmental change is dependent on the maintenance of genetic diversity, and as such, quantifying diversity is central to combating ongoing widespread reductions in biodiversity. With the advent of next‐generation sequencing, several methods now exist for resolving fine‐scale population structure, but the comparative performance of these methods for genetic assignment has rarely been tested. Here, we evaluate the performance of sequenced microsatellites and a single nucleotide polymorphism (SNP) array to resolve fine‐scale population structure in a critically important salmonid in north eastern Canada, Arctic Charr (*Salvelinus alpinus*). We also assess the utility of sequenced microsatellites for fisheries applications by quantifying the spatial scales of movement and exploitation through genetic assignment of fishery samples to rivers of origin and comparing these results with a 29‐year tagging dataset. Self‐assignment and simulation‐based analyses of 111 genome‐wide microsatellite loci and 500 informative SNPs from 28 populations of Arctic Charr in north‐eastern Canada identified largely river‐specific genetic structure. Despite large differences (~4X) in the number of loci surveyed between panels, mean self‐assignment accuracy was similar with the microsatellite loci and the SNP panel (>90%). Subsequent analysis of 996 fishery‐collected samples using the microsatellite panel revealed that larger rivers contribute greater numbers of individuals to the fishery and that coastal fisheries largely exploit individuals originating from nearby rivers, corroborating results from traditional tagging experiments. Our results demonstrate the efficacy of sequence‐based microsatellite genotyping to advance understanding of fine‐scale population structure and harvest composition in northern and understudied species.

## 1 INTRODUCTION

Climate change is having devastating effects on global biodiversity, through accelerated species extinction (Urban, 2006), shifts in distributions and geographic ranges (Chen, Hill, Ohlemüller, Roy, & Thomas, 2011) and reductions of genetic diversity (Pauls, Nowak, Balint, & Pfenninger, 2013). Accordingly, conservation efforts increasingly require rapid and efficient methods for documenting and conserving diversity (Telfer et al., 2015). Central to these efforts is the maintenance of intraspecific diversity, the most fundamental aspect of biodiversity (May, 1994), which can buffer against environmental change (Maestre et al., 2012; Oney, Reineking, O'Neill, & Kreyling, 2012) and promote ecosystem functioning (Raffard, Santoul, Cucherousset, & Blanchet, 2019). However, the conservation of intraspecific diversity requires an understanding of fine‐scale population structure that is often lacking in species with limited genomic resources. Several next‐generation sequencing methods exist for surveying genome‐wide markers that can enable the resolution of fine‐scale structure (e.g., Baird et al., 2008; Bradbury et al., 2018; Davey et al., 2011), but the comparative utility of these methods remains largely unevaluated.

Past studies have relied on microsatellites or single nucleotide polymorphisms (SNPs) for resolving fine‐scale genetic structure, but recent studies increasingly favour SNPs because small numbers of microsatellite loci may lack sufficient power for detection (Putman & Carbone, 2014). However, on a per‐locus basis, multiallelic microsatellites may be more informative than biallelic SNPs (e.g., Hess, Matala, & Narum, 2011), and the recent development of sequence‐based protocols and software for microsatellite genotyping (Zhan et al., 2013) has significantly increased the number of alleles that may be assayed per amplicon compared with SNPs. These advances in microsatellite genotyping have increased the spatial resolution of population structure in a variety of species (e.g., Bradbury et al., 2018; Darby, Erickson, Hervey, & Ellis‐Felege, 2016; Lepais et al., 2019) and offer new opportunities for fisheries and wildlife management.

Arctic Charr (*Salvelinus alpinus*), the most northerly distributed freshwater fish species, exhibits extensive morphological, ecological and behavioural variation across its range (Christensen, Rondeau, et al., 2018; Klemetsen, 2010; Loewen, Gillis, & Tallman, 2010; Reist, Power, & Dempson, 2013) and represents a valuable cultural, economic and ecological resource in Canada. Despite extensive work in genetic stock identification for many important salmonid fisheries, genetic data are limited for Arctic Charr in Newfoundland and Labrador (but see Bernatchez, Dempson, & Martin, 1998), a region that has supported one of the oldest and most productive Arctic Charr fisheries in Canada (landings of 200 t per year in late 1970s) (Andrews & Lear, 1956; Dempson, Shears, Furey, & Bloom, 2008; DFO, 2001). This lack of genomic resources, and basic knowledge of population structure, hinders effective management of the mixed stock fishery and is especially problematic since Arctic Charr may face increased harvest pressure from climate‐induced reductions in sea ice and increased access in the north (Steiner et al., 2018).

Here, we use a novel study of Arctic Charr spatial genetic structure in north‐eastern Canada to compare the performance of genome‐wide microsatellites and highly informative SNPs for population identification and genetic assignment. Specifically, our goals were to (a) compare the scale of population structure using sequenced microsatellites and a SNP array, (b) demonstrate the utility of a genome‐wide panel of sequenced microsatellites to quantify contributions to a mixed stock harvest and the spatial scale of exploitation and (c) compare the geographic scale of genetic individual assignments with a long‐term tagging dataset. Our study is the first to evaluate the efficacy of sequence‐based microsatellite genotyping for population identification, genetic assignment and estimates of dispersal in a northern fish species and has broad implications for the management of other vital understudied northern species.

## 2 METHODS

### 2.1 Sample collection and genotyping

**Figure 1:**
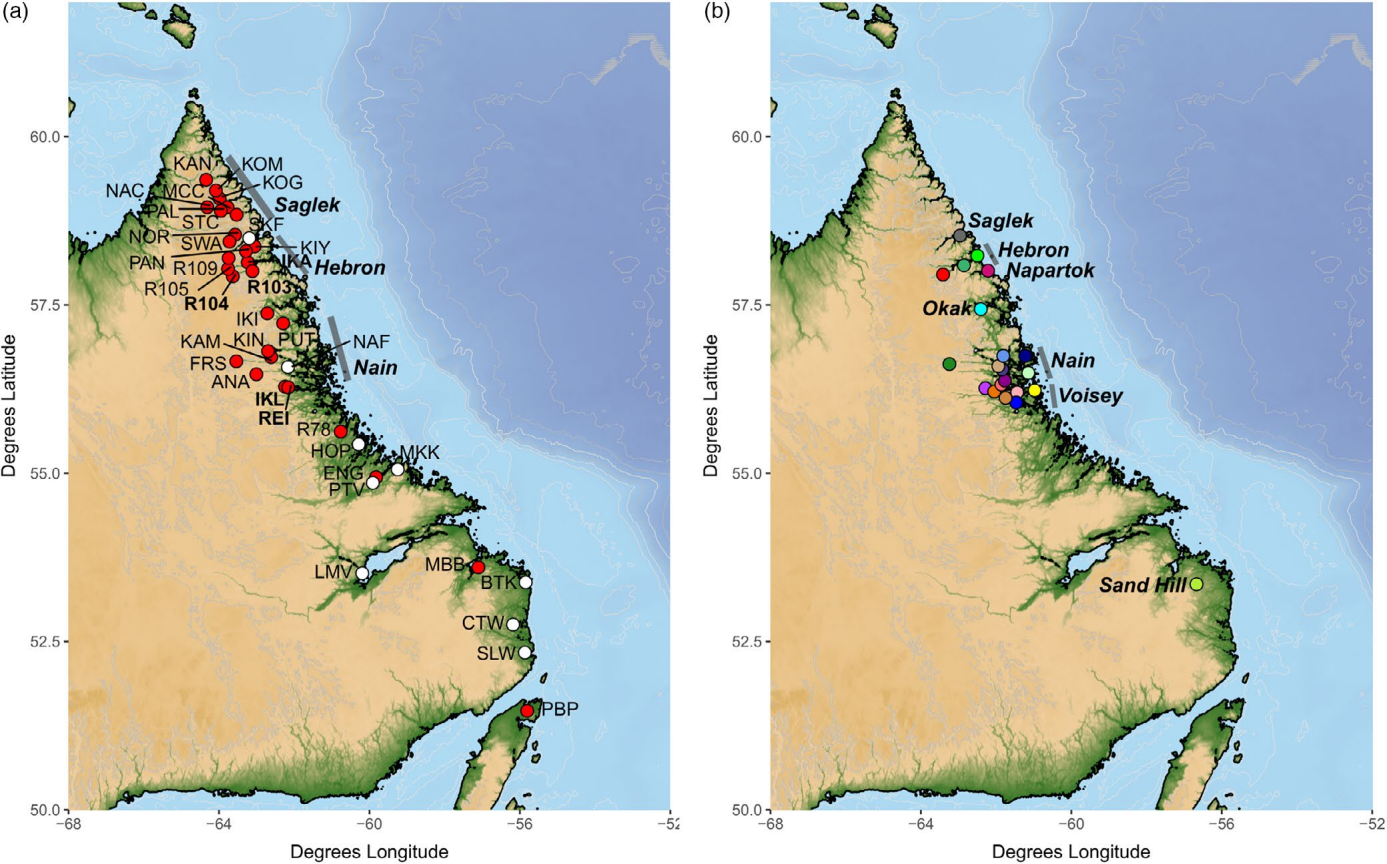
Sampling locations in Newfoundland and Labrador, Canada. (a) Baseline samples (red) and fishery samples (white). Bold text represents populations that clustered into single reporting groups for assignment, and grey lines mark regional areas. (b) Tagging sites (coloured circles correspond to Figure 6) with grey lines delineating general fishing locations

We collected 915 juvenile baseline samples for microsatellite and SNP genotyping from 28 rivers in northern Newfoundland and Labrador between 2005 and 2017 (Figure 1; Table S1). Additional rivers in southern Labrador were surveyed for Atlantic Salmon (*Salmo salar*) in 2016, but no Arctic Charr were encountered (Table S2). Electrofishing provided the majority of samples, but counting fences and angling supplemented collections in Muddy Bay Brook (MBB), English River (ENG) and River 78 (R78). We focused Arctic Charr sampling most intensively on the more abundant and culturally relevant populations in northern Labrador, compared to less abundant southern populations where Arctic Charr are often replaced by Atlantic Salmon and Brook Trout (*Salvelinus fontinalis*) (Andrews & Lear, 1956; Black, Dempson, & Bruce, 1986; Dempson & Green, 1985). An additional 996 individuals of potentially mixed stock origin were collected from nine coastal fisheries in Labrador in 2017 and 2018. All river and fishery acronyms are available in Table S1. We used shore‐set surface gill nets to collect fish from all commercial and food, social and ceremonial (FSC) fisheries, except for Saglek Fjord (SKF) where we used angling. Fin clips from fish were preserved in either 95% ethanol or RNA*later* (ThermoFisher Scientific), prior to extracting DNA using the Qiagen DNeasy 96 Blood and Tissue extraction kit (Qiagen) following manufacturer's guidelines, and quantifying with either Qubit (ThermoFisher Scientific) or QuantIT PicoGreen (Life Technologies).

We identified microsatellite loci using the *Salvelinus* reference genome from Christensen, Rondeau, et al. (2018) and designed oligonucleotides using MSATCOMMANDER v 1.0 (Faircloth, 2008). Microsatellite loci were representative of all available linkage groups (*n* = 37) and comprised dinucleotide loci with 20 repeats and trinucleotide loci with 10–14 repeats. We tested 384 loci (219 dinucleotide and 165 trinucleotide) in pools of 22–23 loci per multiplex PCR in eight individuals to identify useful microsatellites following the protocol of Zhan et al. (2013). Sequencing of microsatellite libraries was conducted on an Illumina MiSeq using 150 cycle v3 reagent kits, and libraries were loaded at 15 pmol concentration. Following sequencing, we used MEGASAT software (Zhan et al., 2013) to demultiplex loci and score microsatellite alleles, setting minimum depth (per sample per locus) at 50 reads; alleles with <50 reads (poor amplification) were not called. Examination of histogram outputs (depth vs. allele size) from MEGASAT confirmed allele scores, and we adjusted scores where necessary. Following initial screening and rejection of poor loci, we selected a final panel of 111 microsatellite loci (45 dinucleotide, 66 trinucleotide) for further genotyping of all DNA samples in five multiplex pools of 21–25 loci per pool (Table S3). Given the potential for species misidentification and/or interspecific hybridization, we used the microsatellite loci and NewHybrids in the R package *parallelnewhybrid* (Wringe, Stanley, Jeffery, Anderson, & Bradbury, 2017), with 100,000 sweeps and a burn‐in of 50,000 to detect and remove Brook Trout and putative Brook Trout/Arctic Charr hybrids from southern populations. In order to assess quality of loci for assignment, we calculated pairwise *F*_ST_ (Weir & Cockerham, 2017) and per‐locus *F*_ST_ for the microsatellite and SNP panels using the *hierfstat* package in R (Goudet & Jombart, 2015). We also used the microsatellite data for pairwise relationship inference in the *CKMRsim* package (Anderson, https://doi.org/10.5281/zenodo.820162) in R to check for family structure. This program calculates probabilities of related and unrelated genotype pairs and computes log‐likelihood ratios comparing a hypothesis of related versus unrelated (Baetscher, Clemento, Ng, Anderson, & Garza, 2018). When assessing the distribution of these log‐likelihood ratios against a false‐positive threshold, populations with ratios exceeding this threshold are expected to contain related individuals. We constructed a neighbour‐joining tree with Cavalli‐Sforza and Edwards distance and 1,000 bootstrap replicates in the *ape* v5.3 (Paradis & Schliep, 2019) and *poppr* (Kamvar, Tabima, & Grünwald, 2014) packages in R and performed principal coordinates analyses (PCoA) in the *ade4* package (Dray & Dufour, 2007) in R.

Single nucleotide polymorphism genotyping used an 87k array following methods by Nugent et al. (2019), filtering for minor allele frequency (0.01) and missing data (>0.05) in PLINK (Purcell et al., 2007) to yield a total of 16,431 polymorphic SNPs. Using all SNPs, we constructed a neighbour‐joining tree with Nei's distance and 1,000 bootstrap replicates in the *StAMPP* (Pembleton, Cogan, & Forster, 2013), *ape* v5.3 (Paradis & Schliep, 2019) and *poppr* (Kamvar et al., 2014) packages in R and performed principal coordinates analyses (PCoA) in the *ade4* package (Dray & Dufour, 2007) in R. Prior to baseline analysis, we selected a panel of highly informative SNPs. *F*_ST_ ranking employed all 16,431 SNPs using the *genepop_toploci* function in the *genepopedit* package in R with an *F*_ST_ threshold of 0.05 and a linkage disequilibrium threshold of 0.2 (Stanley, Jeffery, Wringe, DiBacco, & Bradbury, 2019). SNP selection used a subset of 33% of individuals as a training set. We then assembled the baseline using all individuals but assessed self‐assignment and simulation accuracy and efficiency with a holdout set that excluded individuals from the training set, following methods by Anderson, Waples, and Kalinowski (2008), Anderson (2010) and Sylvester et al. (2015).

### 2.2 Baseline analysis

For baseline analysis with microsatellites and SNPs, we initially identified each river sample as a unique reporting group, revising these reporting groups using an iterative approach. Self‐assignment and simulations assessed the power of assignment to these reporting groups using a leave‐one‐out method in the R package *rubias* (Anderson, 2017; Anderson et al., 2008). Following initial assignment back to reporting groups, we compared a range of actual to simulated proportions across 100 replicates of simulated mixtures, each with 500 fish. We then conducted 100% simulations, deriving 100% of the individuals from one reporting group, using 50 replicates of 100 fish drawn from a flat Dirichlet distribution. Self‐assignment and simulation analyses were conducted for both the microsatellite and SNP panels. Here, we calculated accuracy as the number of correctly assigned individuals divided by the total number assigned to the reporting group and efficiency as the number of correctly assigned individuals divided by the total number known a priori for that reporting group (Bradbury et al., 2018; Vähä & Primmer, 2017). Given instances of low (<50%) assignment accuracy for individual rivers, we revised the baseline using clustering analysis and geographic proximity, to cluster multiple rivers into a single reporting group. Lumping populations increased accuracy when they formed sister groups in the NJ analysis and occurred in close geographic proximity (<15 km). We repeated self‐assignment and simulations again on the revised reporting groups until we reached minimum accuracy.

### 2.3 Mixed stock fishery analysis

We conducted a mixed stock fishery analysis with a Bayesian approach in *rubias* (Anderson, 2017; Moran & Anderson, 2018), using individuals retrieved from mixed stock fisheries. For individual assignments and proportions of each mixed stock fishery, we used 20,000 MCMC iterations, discarding the first 1,000 iterations as burn‐in. We estimated the proportion contributed from each reporting group to the mixed stock fishery and computed 95% credible intervals from the MCMC traces of mixing proportions; we excluded reporting groups with credible intervals encompassing zero. We also examined the relationship between the number of fish assigned to a reporting group and the distance of that reporting group from each of nine fisheries. For fisheries with more than two contributing reporting groups, linear regression in R determined the relationship between drainage area and the relative proportion of reporting groups contributing to the mixed stock fishery. We calculated the least‐cost distance between sites with the *lc.dist* function in *marmap* (Pante & Simon‐Bouhet, 2013) and then plotted the number of individuals assigned to a reporting group against the geographic distance between the assigned reporting group and mixed stock fishery.

### 2.4 Analysis of tagging experiments

Arctic Charr tagging and recapture spanned 21 sites from eight regional locations in Labrador between 1974 and 2003, herein referred to as “stocks” (Figure 1). Fish were caught during the spring outmigration by angling or in shore‐set surface gill nets and tagged using Carlin tags with double stainless‐steel thread (Dempson & Kristofferson, 1987; Dempson, Shears, Furey, & Bloom, 2004). Upstream migrating Charr were recaptured at fish counting fences in Fraser River and Ikarut River, or in shore‐set surface gill nets during the summer fishery. We calculated the least‐cost distance between tagging and recapture sites with the *lc.dist* function in *marmap* (Pante & Simon‐Bouhet, 2013). Linear regression in R determined the relationship between geographic distance from tagging site and the number of individuals recaptured for each stock. We also estimated the percentage of individuals recaptured from their original tagging (potentially natal) site and compared them to fish recaptured from non‐natal sites. Importantly, the original tagging site may not coincide with the natal river for overwintering individuals, and variation in recapture rates may reflect a difference in recapture effort among sites.

## 3 RESULTS

### 3.1 Locus assessment

**Figure 2:**
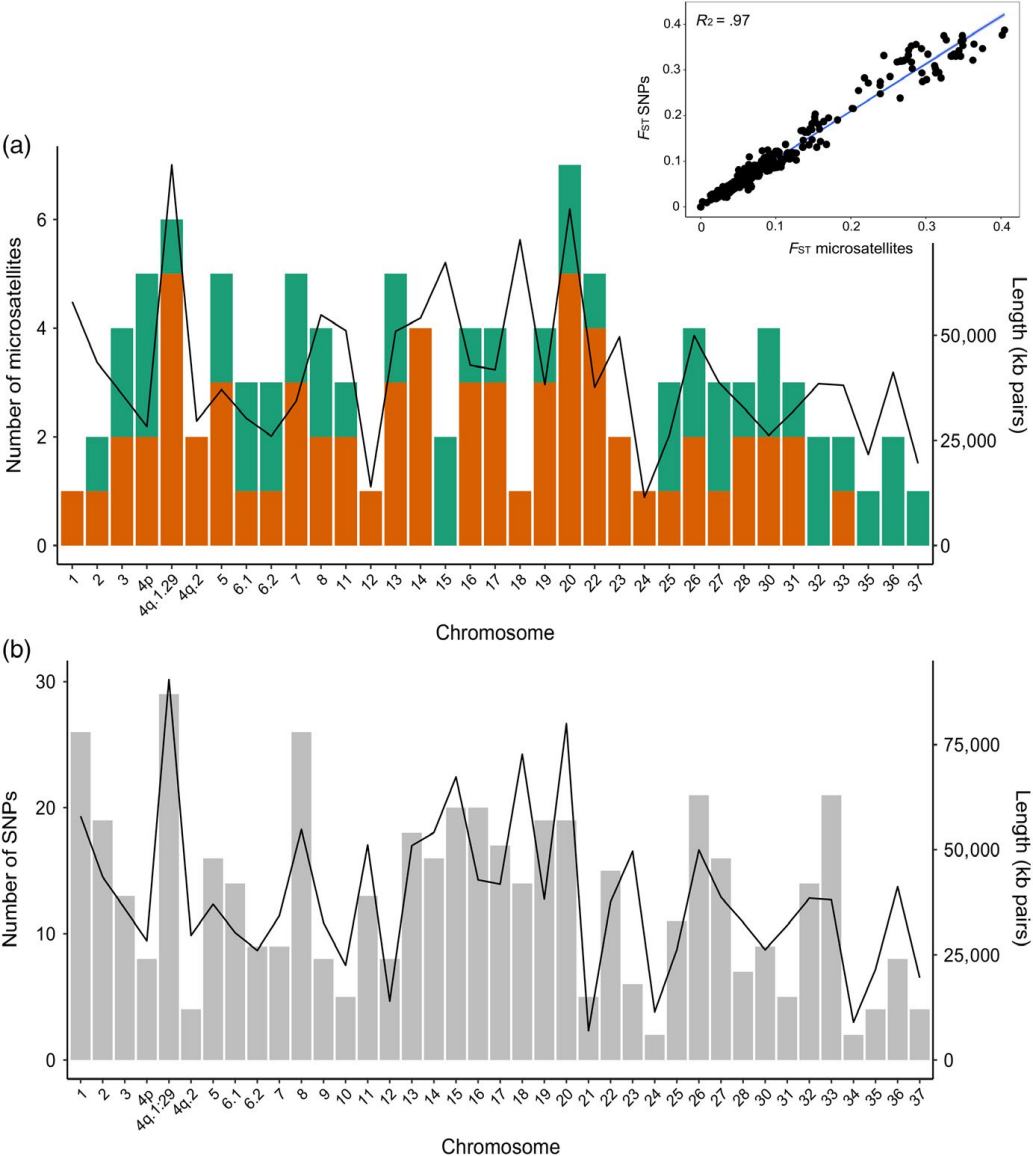
(a) Distribution of dinucleotide (green) and trinucleotide (orange) microsatellite loci (inset: linear regression of pairwise *F*_ST_ values from microsatellites and SNPs). (b) *F*_ST_ ranked SNPs across the *Salvelinus* genome, with chromosome length in kilobase pairs (line)

Baseline analysis of 800 individuals from 28 populations in northern Newfoundland and Labrador used a panel of 111 microsatellite loci (45 dinucleotide, 66 trinucleotide) distributed across the genome (Figure 2a), excluding linkage groups 9, 10, 21 and 34, with an average of 3.2 loci per chromosome (range 1–7). This panel identified 807 alleles (mean 7.3 per locus) (Figure S1a) with per‐locus *F*_ST_ ranging from 0.02 to 0.28 (Figure S1b) and a mean pairwise *F*_ST_ of 0.10. We used a reduced set of microsatellite loci (*N* = 106) for mixed stock fisheries analysis in 996 individuals because some loci failed to amplify in all samples. Pairwise *F*_ST_ was highest between Parker's Bay Brook (PBP) in Newfoundland and Kiyuktok Brook (KIY) in northern Labrador with both microsatellite and SNP panels (0.39 and 0.41, respectively) and lowest between Ikarut River (IKA) and River 103 (R103) with microsatellites and R103 and River 104 (R104) with SNPs (0.008 and 0.009, respectively) (Table S4). Relationship inference in CKMRsim with the microsatellite data showed that population KIY contained individuals that were more related than expected by chance (Figure S2). However, putatively related individuals impacted only a single population (KIY), and Waples and Anderson (2006) demonstrated that removing siblings may bias population genetic inference; we therefore retained these individuals for the remainder of the analysis. Subsequent baseline analysis utilized a panel of unlinked, *F*_ST_‐ranked SNPs with 744 individuals and for assessing self‐assignment and simulation accuracy with the holdout dataset. These SNPs were distributed across the genome, averaging 12.8 SNPs per chromosome (range 2–29) (Figure 2b) and a mean pairwise *F*_ST_ of 0.11.

### 3.2 Microsatellite and SNP baseline performance

**Figure 3:**
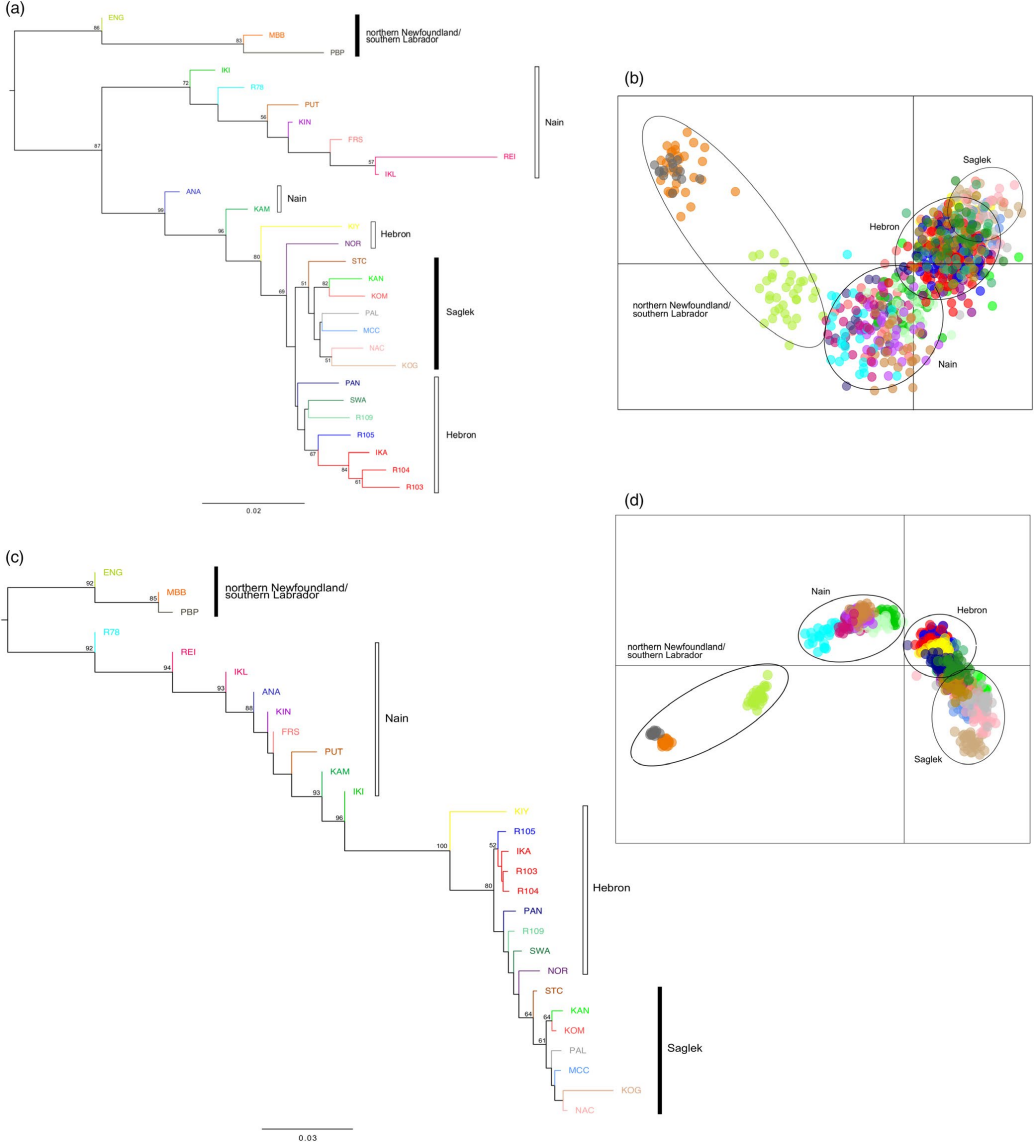
(a) Neighbour‐joining tree (Cavalli‐Sforza and Edwards distance) and (b) principal coordinates analyses (PCoA) performed with microsatellite data. (c) Neighbour‐joining tree (Nei's distance) and (d) principal coordinates analyses (PCoA) performed with SNP data. Branches are coloured by reporting groups, and bootstrap values >50% are provided. Regional groupings are denoted by a bar, with solid bars indicating monophyly and open bars indicating non‐monophyly

**Figure 4:**
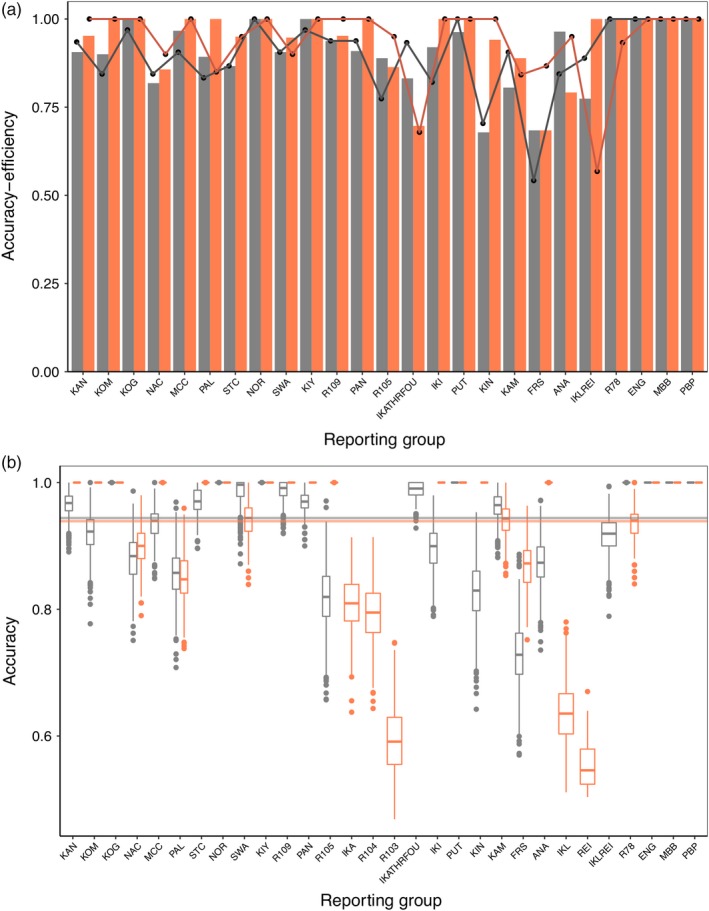
Comparing individual assignment and mixture simulation results in *rubias* between microsatellite and SNP panels. (a) Individual assignment accuracy (bars) and efficiency (line) of Arctic Charr to 25 reporting groups based on microsatellites (grey) and SNPs (orange). For SNP data, accuracy and efficiency were averaged across populations that clustered into reporting groups for microsatellite assignment. (b) Accuracy of 100% mixture simulations for individuals with >50% probability from microsatellite (grey) and SNP (orange) datasets, with solid lines representing mean accuracy for each dataset. Both individual (SNP) and clustered (microsatellite) reporting groups are shown, arranged by decreasing latitude

Initial baseline analysis assigned each river to a unique reporting group, but we subsequently combined some rivers into reporting groups, depending on assignment accuracy, geographic proximity and clustering in the NJ tree (Figure 3). The NJ trees and PCoAs for both microsatellites and SNPs recovered fine‐scale structure within broader regional areas, but populations were more resolved in the SNP PCoA and sister groups were better supported in the microsatellite NJ tree (Figure 3). Northern Newfoundland/southern Labrador and Saglek regions were recovered as monophyletic in both NJ trees, but the Nain and Hebron regions were recovered as paraphyletic (Figure 3). Genetic assignment analysis with the microsatellite panel supported clustering of three rivers in the Hebron area (IKA, R103, R104) and two rivers in the Nain area (IKL, REI) into single reporting groups (IKAFOUTHR and IKLREI), respectively. These clusters reduced the number of reporting groups from 28 to 25, although less than 15 km separated these rivers, and they formed sister groups in the NJ tree. After conducting self‐assignment analysis with SNP panels ranging in size from 100 to 1,000 SNPs, we found a strong correlation between mean accuracy and panel size (*R* = .86), with mean accuracy ranging from 77.9% to 94.6%. A final panel of 500 SNPs chosen for downstream analysis represents an optimal trade‐off point between assignment accuracy (>90%) and cost, and previous studies have used similarly sized panels for genetic assignment in salmonids (e.g., Sylvester et al., 2015). The SNP panel supported each river as a unique reporting group for a total of 28 reporting groups. Mean self‐assignment accuracy for the microsatellite and SNP reporting groups differed at 90.5% and 94.1%, respectively, but the differences were insignificant (two‐tailed *t* test; *p* = .18) (Figure 4a). Mean self‐assignment efficiency also differed between microsatellite and SNP datasets at 89.5% and 93.6%, respectively, but were also insignificant (two‐tailed *t* test; *p* = .19) (Figure 4a). Self‐assignment accuracy was >80% for 86.8% of the individuals in the microsatellite dataset and 95.3% of the individuals in the SNP dataset. The 100% simulations revealed similarly high accuracy to reporting group but mean accuracy differed significantly, though by a small magnitude between the microsatellite (94.4%) and SNP (93.9%) panels (two‐tailed *t* test; *p* < .001) (Figure 4b). Confidence intervals for the 100% simulations were wider in the microsatellite dataset than the SNP dataset for some overlapping reporting groups (Figure 4b), with lowest accuracy in rivers in the Hebron and Nain regions across both datasets and for both self‐assignment and simulations. Despite a 1:1 relationship between true and simulated proportions for most microsatellite and SNP reporting groups in the leave‐one‐out simulations, we found evidence of upward bias in IKATHRFOU with the microsatellite dataset and upward bias in ANA and downward bias in IKL and REI with the SNP dataset (Figure S3). Because five times fewer microsatellite amplicons produced similarly high accuracy (>90%) for self‐assignment and simulations, subsequent mixed stock fishery analysis used this dataset.

### 3.3 Contributions to mixed stock fisheries

**Figure 5:**
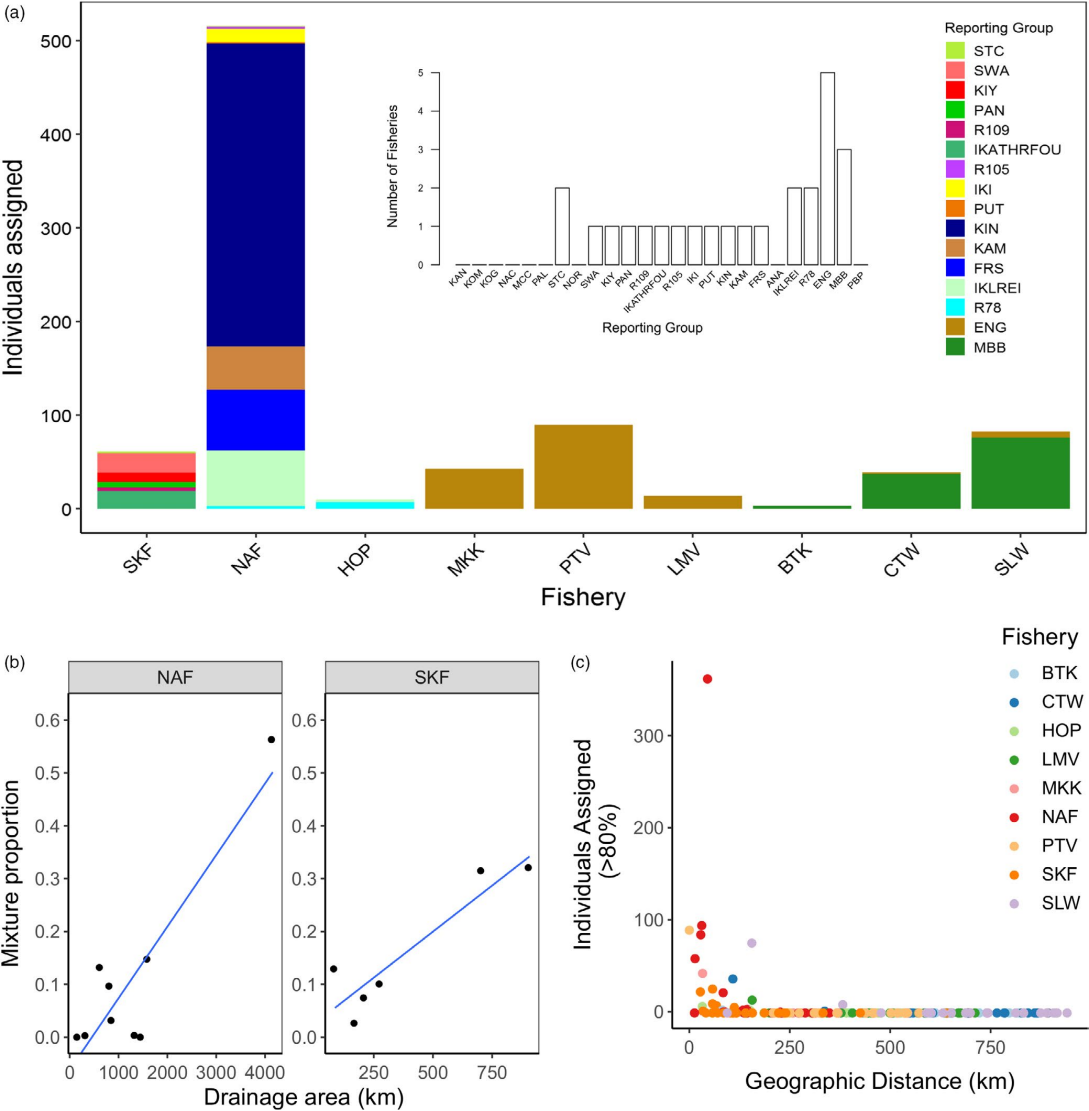
Genetic mixed stock fishery analysis. (a) Number of individuals from each reporting group that assigned to a mixed stock fishery with greater than 80% probability (inset: number of mixed stock fisheries that each reporting unit contributes to). Populations are ordered by decreasing latitude. (b) Linear regression of the relative proportion of multiple reporting groups contributing to the NAF and SKF mixed stock fisheries and drainage area for those reporting groups (*R* ^2^ = .44, *p* = .004). (c) Number of individuals assigned to a reporting group from each of nine mixed stock fishery samples and the geographic distance between the assigned reporting group and mixed stock fishery

The microsatellite baseline established a total of 25 reporting groups, and we estimated their contributions to nine mixed stock fisheries along the coast of Labrador (see Figure 5). A total of 876 individuals assigned to reporting groups with greater than 80% probability in the fisheries analysis, representing 87.8% of the total dataset. Individuals from the northern‐most SKF assigned to multiple reporting groups in the Saglek region with the majority assigning to Southwest Arm (SWA), and individuals from the Nain fishery (NAF) also assigned to multiple reporting groups in the Nain region, with the majority assigning to the Kingurutik River (KIN). Individuals from the Hopedale fishery (HOP) assigned to the IKLREI and R78 reporting groups, whereas individuals from the Lake Melville (LMV), Makkovik (MKK) and Postville (PTV) fisheries assigned entirely to the ENG reporting group. Charr caught in the Charlottetown (CTW) fishery in southern Labrador assigned to both ENG and MBB, with most individuals assigning to the latter, whereas the Black Tickle (BTK) and St. Lewis (SLW) fisheries assigned entirely to the MBB reporting group. Individuals sampled from the Saglek and Nain fisheries assigned to multiple (>2) reporting groups, with a positive relationship between the proportion of individuals assigned to a reporting group and the drainage size of that reporting group (*R* ^2^ = .44, *p* = .004) (Figure 5a, 5b). Fisheries in southern Labrador only assigned to one or two reporting groups (Figure 5a, Figure S4). Overall, the mixed stock analysis demonstrated that fisheries target nearby rivers, with a 60% probability that individuals originate from within 200 km of the fishery (Figure 5c). When we excluded individuals from southern Labrador, where we lack extensive baseline coverage, we found a 75.6% probability that individuals originated from within 200 km of the fishery.

### 3.4 Spatial patterns of movement

**Figure 6:**
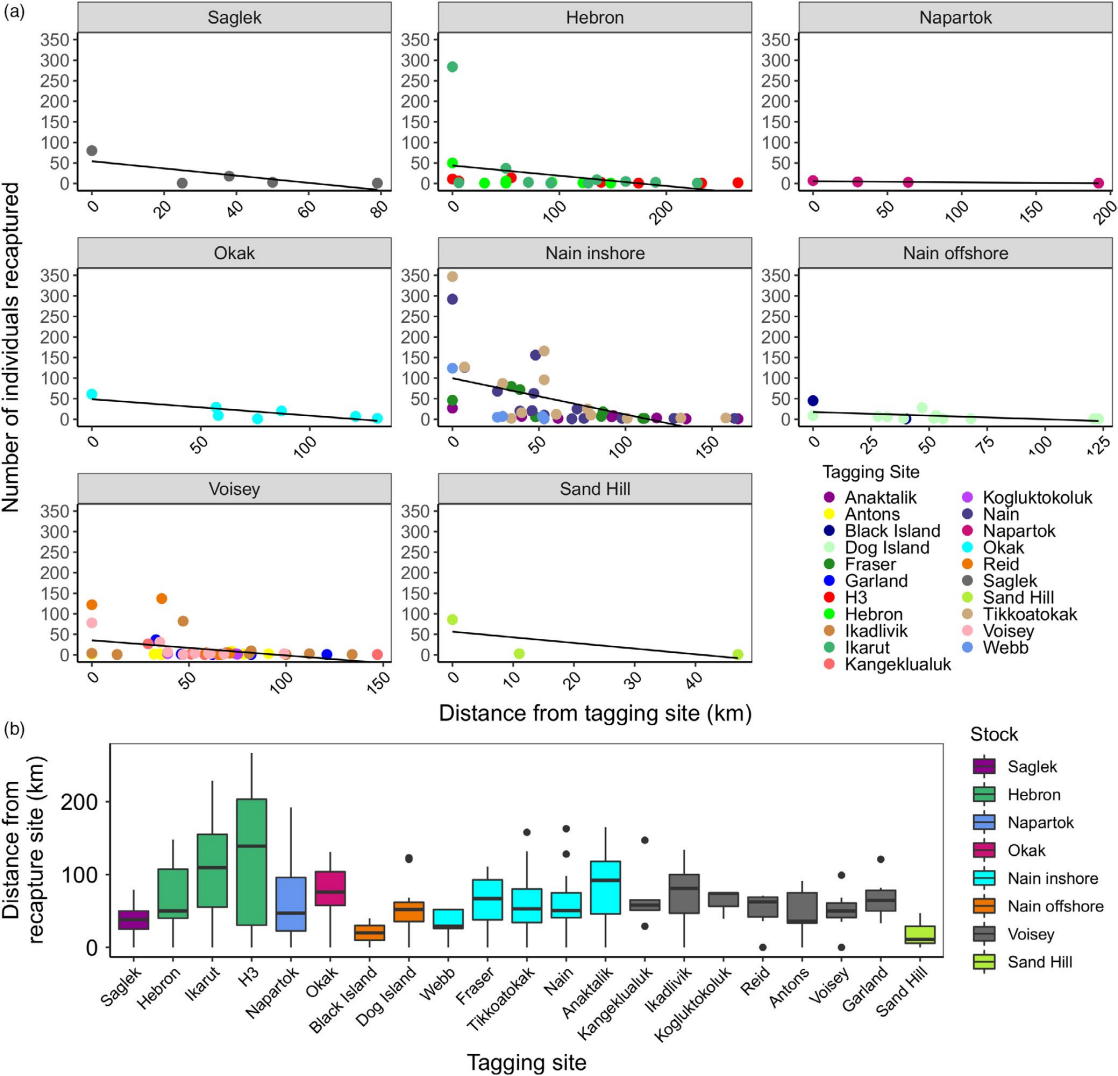
Tag recapture analysis of Arctic Charr in northern Labrador. (a) Linear regression of the number of individuals recaptured and their distance from the tagging site (*R* ^2^ = .14, *p* ≤ .0001). (b) Distribution of distances between tagging and recapture site for individuals from eight fishery stocks. Both panels order stocks by decreasing latitude

We also incorporated tagging data from eight fishing locations across Labrador with data from 15,950 tagged fish and 3,712 recaptures (Table S5; Figure S5), with most recaptures originating from a fishery. We calculated least‐cost distance between tagging and recapture sites and detected a negative, though weak (R^2^ = 0.14, *p* < .0001) relationship between the number of individuals recaptured and distance from tagging site (Figure 6a). Our results confirm limited movement at sea in Arctic Charr, with most individuals recaptured within 100 km of their tagging site (Figure 6b); the greatest distance between tagging and recapture was 267 km. Mean distance between tagging and recapture site was highest in the H‐3 (124.9 km) and IKA (106.2 km) populations in the Hebron region. The percent of fish recaptured from their region of tagging ranged from 46.7% in Napartok to 100% in Sand Hill, with a mean recapture rate of 78.7% (Table S5).

## 4 DISCUSSION

### 4.1 Comparison of sequenced microsatellites and SNPs

Effective fisheries management and population persistence hinge upon understanding population structure and stock composition (Hilborn, Quinn, Schindler, & Rogers, 2003; Ruzzante et al., 2006; Schindler et al., 2010), particularly in northern species facing increased exploitation and growing pressure from climate change. Yet the paucity of genomic resources for many northern species and growing suite of potential methods make identifying best practices difficult. Here, we evaluated sequence‐based microsatellite genotyping and a highly targeted SNP panel to resolve fine‐scale structure in a northern fish, the Arctic Charr. The results demonstrate the comparable power of sequence‐based microsatellites and highly targeted SNP panels for genetic assignment and population identification. In fact, the sequenced microsatellite panel genotyped 7X the number of alleles per amplicon in comparison with a highly selected SNP panel and achieved similar assignment accuracy. Furthermore, this work demonstrates the utility and high efficiency of panels of sequenced microsatellites through application to mixed stock fishery analyses and comparison with long‐term tagging experiments which both indicate restricted dispersal within coastal fisheries. Our results build directly on recent studies developing panels of sequence‐based microsatellites in salmonids (Bradbury et al., 2018) and other taxa (Darby et al., 2016; Neophytou et al., 2018), the development of software for sequence‐based microsatellite genotyping (e.g., Zhan et al., 2013) and clearly illustrates the large potential for sequence‐based microsatellite genotyping to other northern fish and/or understudied species.

Sequence‐based microsatellite genotyping (e.g., Zhan et al., 2013) allows the interrogation of larger numbers of loci than traditional electrophoretic approaches and has improved fine‐scale inferences of population structure and individual assignment accuracy in salmonids (Bradbury et al., 2018), muskrats (Darby et al., 2016), trees (Neophytou et al., 2018) and across multiple phyla (Lepais et al., 2019). The comparably low cost of $0.06 (CD) per microsatellite locus here compares favourably with the cost reported by Bradbury et al. (2018) upon its inception. Our examination of assignment accuracy of the microsatellite panel (>90%) matches the accuracy of the SNP panels tested here and previous studies in northern salmonid populations (e.g., Beacham, McIntosh, & Wallace, 2010; Bradbury et al., 2016; Bradbury et al., 2015; Jeffery et al., 2018) but dramatically increases the spatial resolution from regional groupings to individual river populations. These observations are strikingly similar to previous work comparing SNPs and sequenced microsatellites for Atlantic Salmon in the Labrador region (Bradbury et al., 2018) which also detected largely river scale structure and comparable accuracy between sequenced microsatellites and selected SNP panels. In both cases, comparison of SNPs and sequenced microsatellites demonstrates similar population resolution and assignment accuracies despite a nearly fourfold increase in sequences targeted in the SNP panel, likely attributable to the increased number of alleles detected with the microsatellite panel (e.g., Kalinowski, 2004).

The emerging consensus based on the current analysis and previous comparisons is that large panels of sequenced microsatellites effectively resolve fine‐scale population structure in understudied species and new environments as well as highly targeted panels of SNPs. These panels of sequenced microsatellites can be developed cheaply and quickly either from existing published genomic resources or new sequencing data. In comparison with sequenced microsatellite loci, our results suggest that the main advantage of targeted panels of SNPs is the potential to resolve specific instances of weak divergence such as between adjacent populations. For example, both here and in Sylvester et al. (2015), highly selected SNP panels were required to distinguish extreme fine‐scale differences among Labrador Atlantic Salmon and Arctic Charr populations in close proximity or sharing estuaries. The obvious downside of targeted SNP panels is that the broad applicability of these highly ascertained panels will likely suffer as has been noted repeatedly with the use of SNP arrays. Significant reductions in diversity have been reported using this Arctic Charr SNP array due to ascertainment bias with losses of 75% to 95% of polymorphic loci when applied to specific Canadian populations (Nugent et al., 2019). Accordingly, sequenced microsatellites may provide a highly efficient balance of population resolution, broad applicability and cost in poorly studied species where genomic resources are lacking and ascertainment bias may be an issue.

### 4.2 Population structure in Arctic Charr

The population structure detected in Arctic Charr in Labrador was largely hierarchical and generally similar across methods with both suggesting a latitudinal cline in structure with southern populations being most divergent. Nonetheless in both cases, highly accurate assignment of individuals to most rivers was achievable and clearly suggest Arctic Charr are structured at the river scale, extending earlier genetic studies of anadromous Arctic Charr in the Canadian Arctic and Labrador (Bernatchez et al., 1998; Boguski, Gallagher, Howland, & Harris, 2016; Moore, Harris, Tallman, & Taylor, 2013; Salisbury et al., 2017) and confirming the propensity for homing in anadromous salmonids and reduced dispersal at sea (Dempson & Kristofferson, 1987; Moore et al., 2013). Despite evidence of higher rates of straying among rivers in Arctic Charr than other salmonids (Moore et al., 2013), fine‐scale population structure at the river level in our study suggests that straying fish do not facilitate significant gene flow. This observation aligns with previous findings of mostly non‐breeding individuals utilizing non‐natal habitats for overwintering (Dutil, 1986; Moore et al., 2013), limited gene flow restricted to neighbouring populations (Christensen, Jacobsen, Nygaard, & Hansen, 2018) and the pattern reported in Dolly Varden Charr (*Salvelinus malma*) (e.g., Armstrong & Morrow, 1980). Despite common non‐reproductive straying in Arctic Charr, less information exists regarding their homing patterns, although high levels of genetic differentiation that parallel other salmonid species suggest fine‐scale homing in Arctic Charr. For instance, the mean pairwise *F*_ST_ among populations of Arctic Charr from southern Labrador (*F*_ST_ ~ 0.22) exceeded that of Atlantic Salmon from the same geographic region using a similar number of microsatellites (*F*_ST_ ~ 0.05; Bradbury et al., 2018). Given evidence of high homing fidelity to natal rivers in Atlantic Salmon (King, Kalinowski, Schill, Spidle, & Lubinski, 2001), our results reflect similar levels of homing in Arctic Charr and support conclusions of high rates of natal homing in this species by both Johnson (1980) and Bernatchez et al. (1998).

### 4.3 Application of microsatellite panel—fishery analysis and comparison with tagging data

We further demonstrate the utility of sequence‐based microsatellite loci to disentangle the composition of mixed stock fisheries and spatial scale of movement in Arctic Charr in Labrador. The results of genetic assignment using the microsatellite loci indicate that these fisheries primarily target local populations, with most individuals genetically assigning to rivers within 200 km of the fishery. Comparison of these microsatellite‐based assignments with extensive tagging data allowed independent estimates of movement. The tagging data indicate capture of most individuals within 100 km of their tagging site, corroborating our genetic assignments and past tagging studies of Arctic Charr in other regions of Canada (Gyselman, 1984; Johnson, 1980; Moore, 1975; Morris & Green, 2012; Spares, Stokesbury, Dadswell, O'Dor, & Dick, 2015) and Norway (Finstad & Heggberget, 1992). Regional differences in dispersal and movement patterns may reflect contrasting within‐river distance, or they may also link to prey availability and abundance (Dempson, Shears, & Bloom, 2002), or to availability of suitable overwintering habitats. For example, Dempson and Green (1985) reported that only 20% of fish tagged in Fraser River were recovered there, with others recaptured in nearby Nain and Tikkoatokak Bay, a pattern consistent with our results. Given the high rates of dispersal in non‐spawning individuals (Dutil, 1986; Moore et al., 2013), and the tendency for these individuals to use larger nearby rivers for overwintering (Beddow, Deary, & McKinley, 1998), a portion of the individuals tagged in these rivers likely originated elsewhere, potentially impacting estimates of movement distances. Nonetheless, the similarity in estimates produced here based on both genetic assignment and long‐term tagging experiments support discrete river‐population structure and restricted straying in the marine environment.

Although genetic and tagging‐based estimates were largely congruent, the genetic estimates suggest slightly larger spatial scales of movement than the tagging experiments, even when excluding populations in the south. This difference suggests that genetic‐based estimates may resolve rarer straying events, which may be missed with tagging‐based estimates of movement, further demonstrating the utility of sequenced microsatellites for fisheries applications. However, it is worth noting that the tagging experiments did not necessarily tag fish in natal rivers, and tagging in overwintering rivers near fishery locations could potentially reduce our estimates of movement. Furthermore, the genetic component of our study did not include all anadromous Arctic Charr populations in the region. Although low assignment probability of some individuals may indicate that populations in the region remain unsampled (Waples & Gaggiotti, 1984), our study included most large rivers and known Charr producing rivers in Labrador. Under‐sampling of some regions could explain slightly larger estimates of movement based on the genetic baseline, assuming assignment of individuals to adjacent, but non‐natal rivers. Additional sampling in this region, and particularly better spatial coverage from southern Labrador, may help to refine these genetic‐based estimates of movement and straying. However, the distribution of rivers with Arctic Charr described by Black et al. (1986) indicates only ~10% of rivers known to contain Arctic Charr are located in southern Labrador. Moreover, historically the harvest of Arctic Charr has been centred around the north where 80% of the Arctic Charr are harvested from three stocks (DFO, 2001), with more limited fisheries occurring in southern Labrador. As such, the distribution of samples in our current study accurately reflects both the distribution of the species and the fishery in the region and it is highly unlikely that additional sampling of southern populations would alter the conclusions made here.

## 5 CONCLUSIONS

Our comparison of marker types validates the efficacy of a sequence‐based, genome‐wide microsatellite panel for detecting river‐specific structure in salmonids and for elucidating fine‐scale patterns of exploitation and movement. Our results demonstrate river scale population structure and limited gene flow in Arctic Charr, despite evidence of some straying among rivers. We show general agreement between both genetic assignment based on sequenced microsatellites and long‐term tagging data suggesting that coastal fisheries generally exploit individuals within 100–200 km of their natal river. The current study demonstrates the efficacy of sequence‐based microsatellite genotyping to advance understanding of fine‐scale population structure and harvest composition in northern and understudied species and provides a valuable tool for fisheries management and conservation.

## CONFLICT OF INTEREST

None declared.

## Data Availability

Rubias and Genepop data files for this study are available in the Dryad repository at: https://doi.org/10.5061/dryad.b5mkkwh8q.
