# Supplementary figures and images for: Resolving fine‐scale population structure and fishery exploitation using sequenced microsatellites in a northern fish
Source: Evol Appl. 2020 Feb 20;13(5):1055–68. doi: 10.1111/eva.12922 (PMC7232759; doi:10.1111/eva.12922)

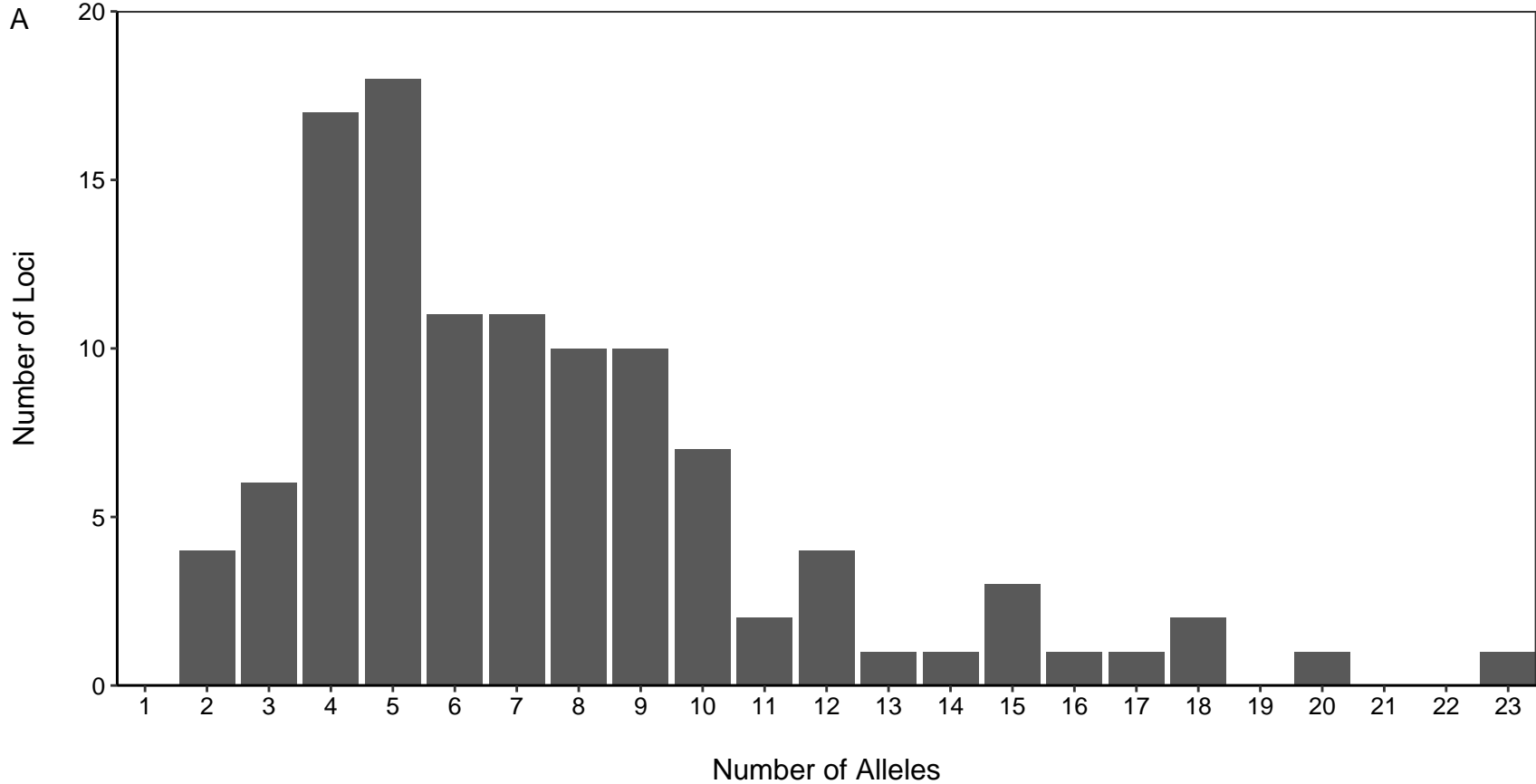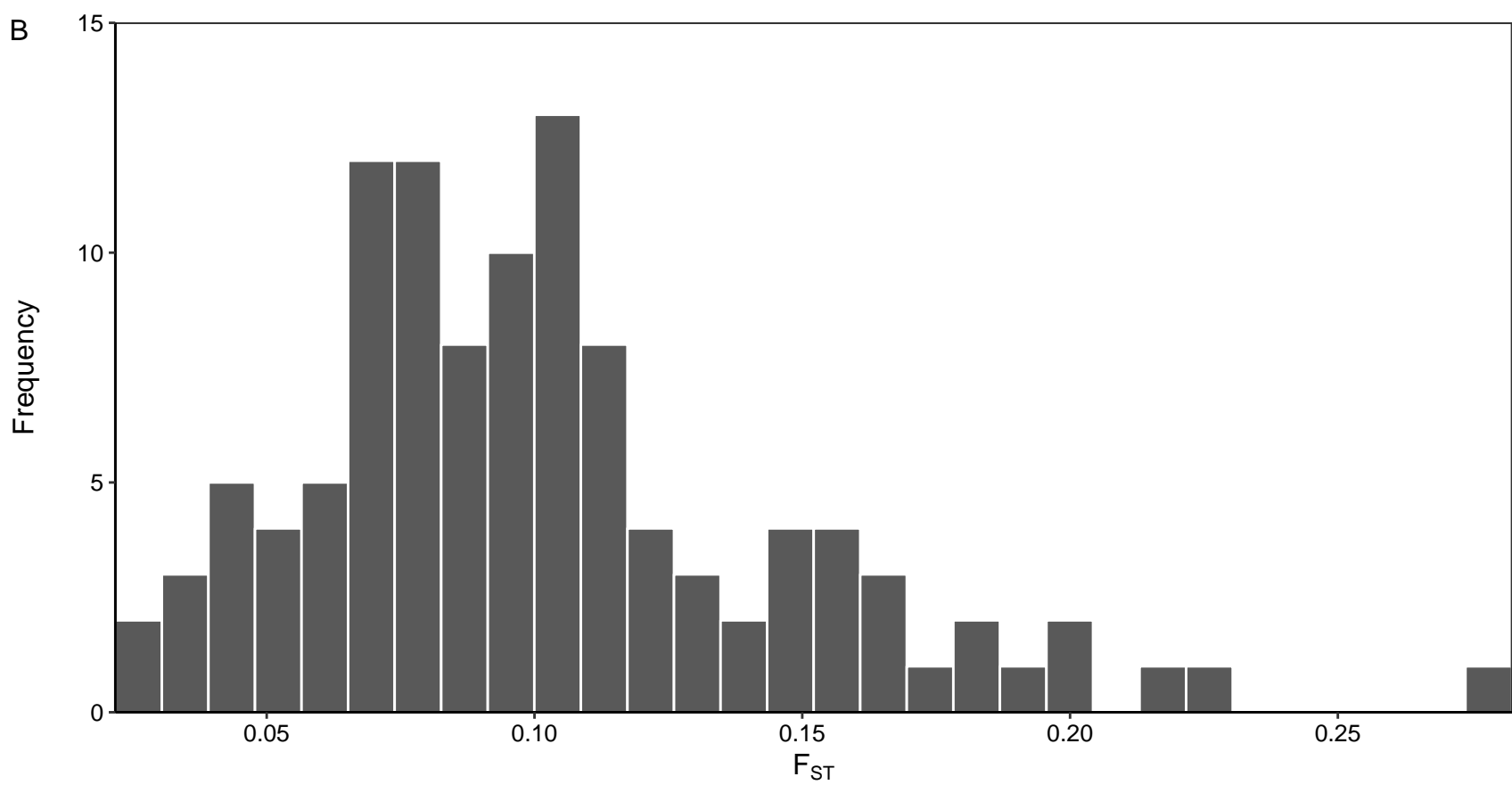

Supplement: Supplementary file 1 [file EVA-13-1055-s001.pdf]

Log L Ratio

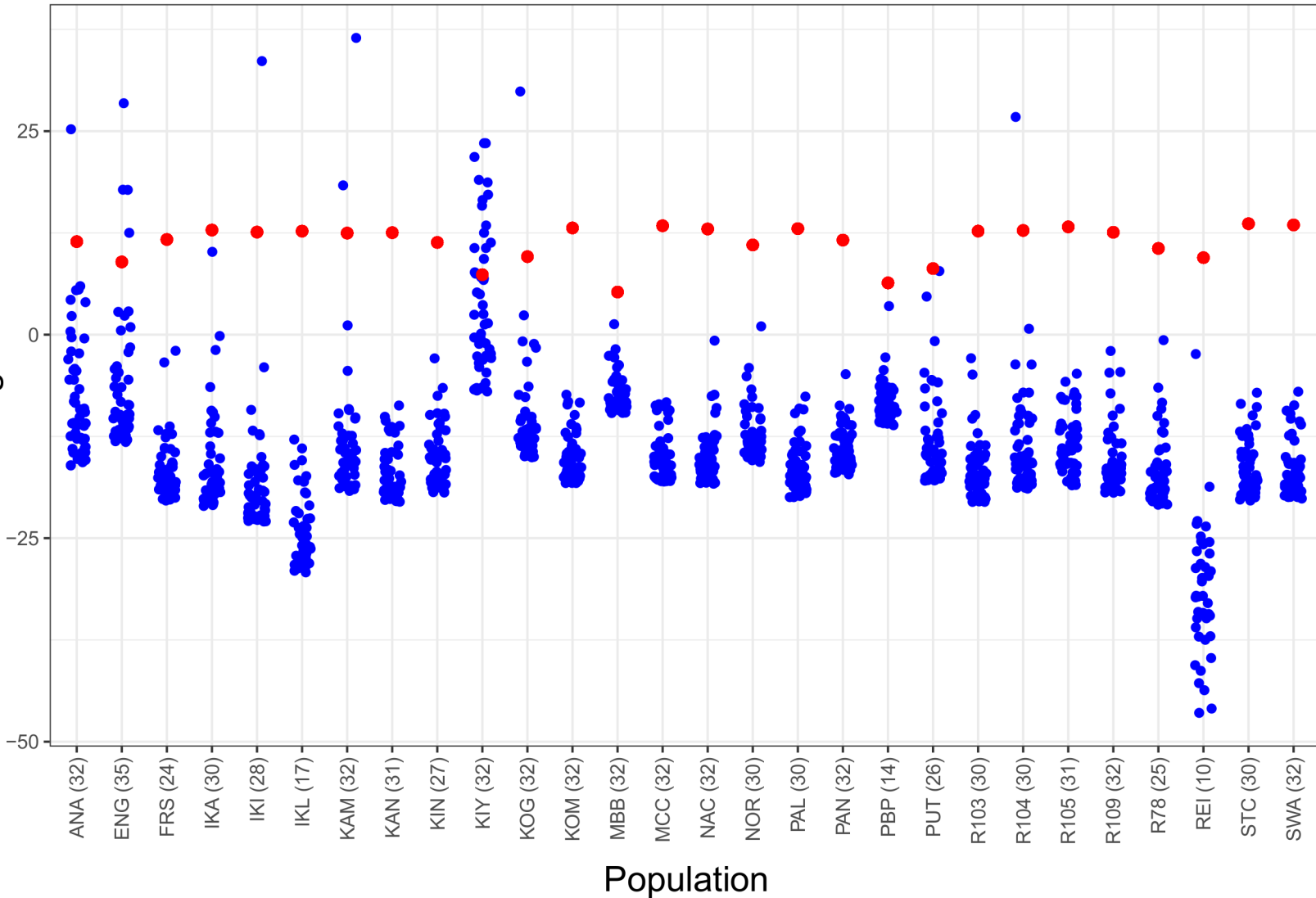

Supplement: Supplementary file 2 [file EVA-13-1055-s002.pdf]

Simulated Mixing Proportion

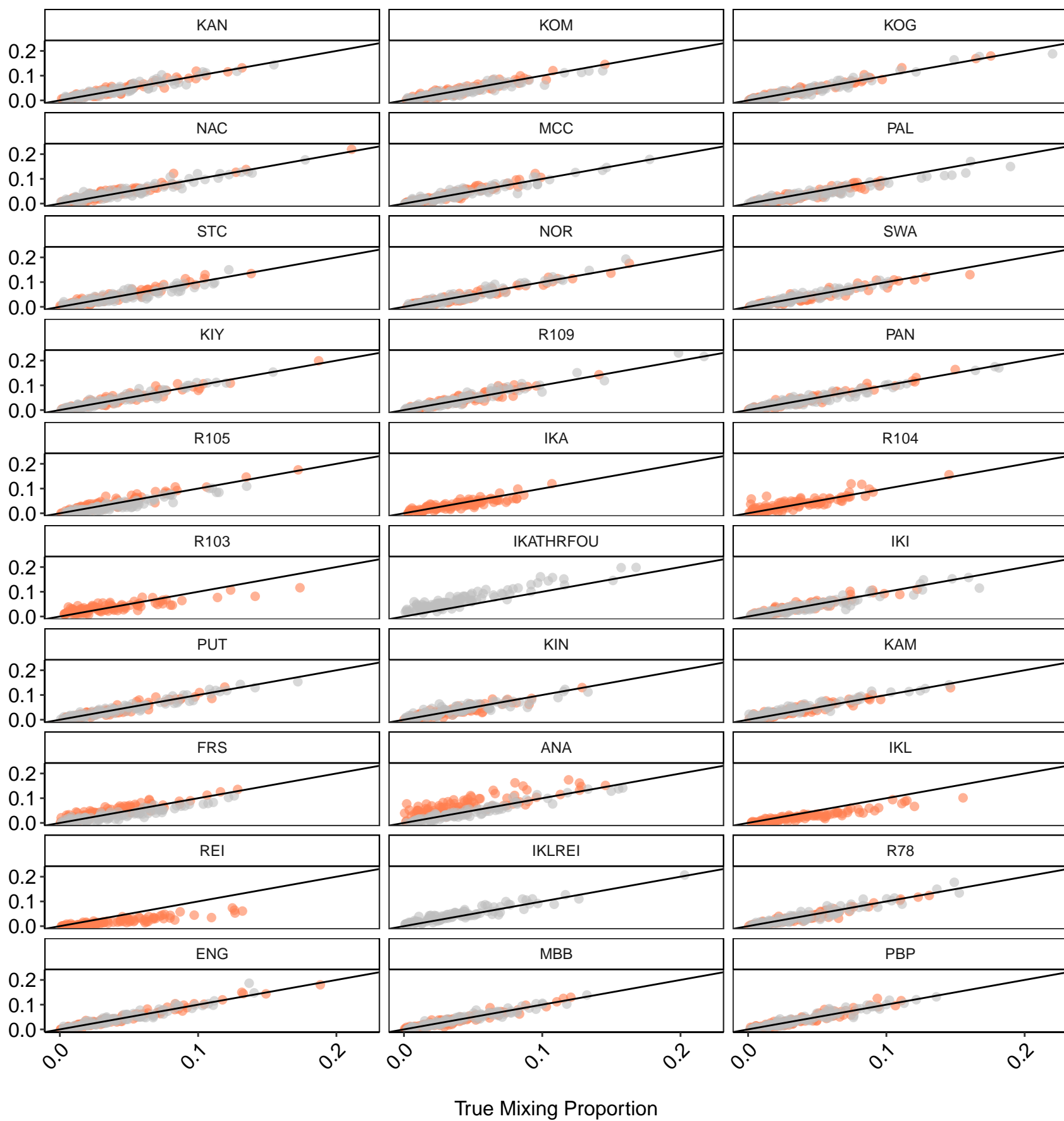

Supplement: Supplementary file 3 [file EVA-13-1055-s003.pdf]

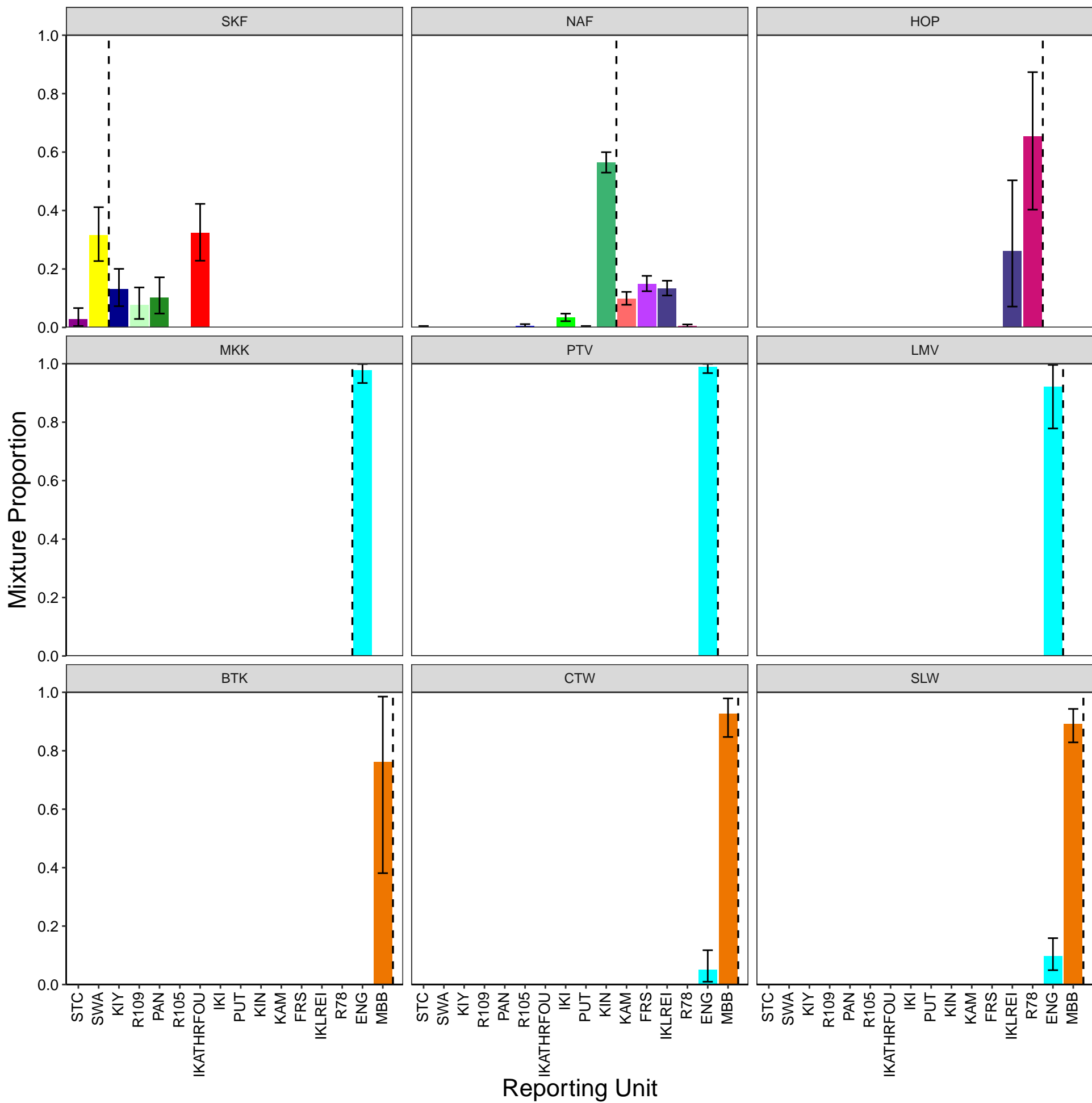

Supplement: Supplementary file 4 [file EVA-13-1055-s004.pdf]

Number of individuals recaptured

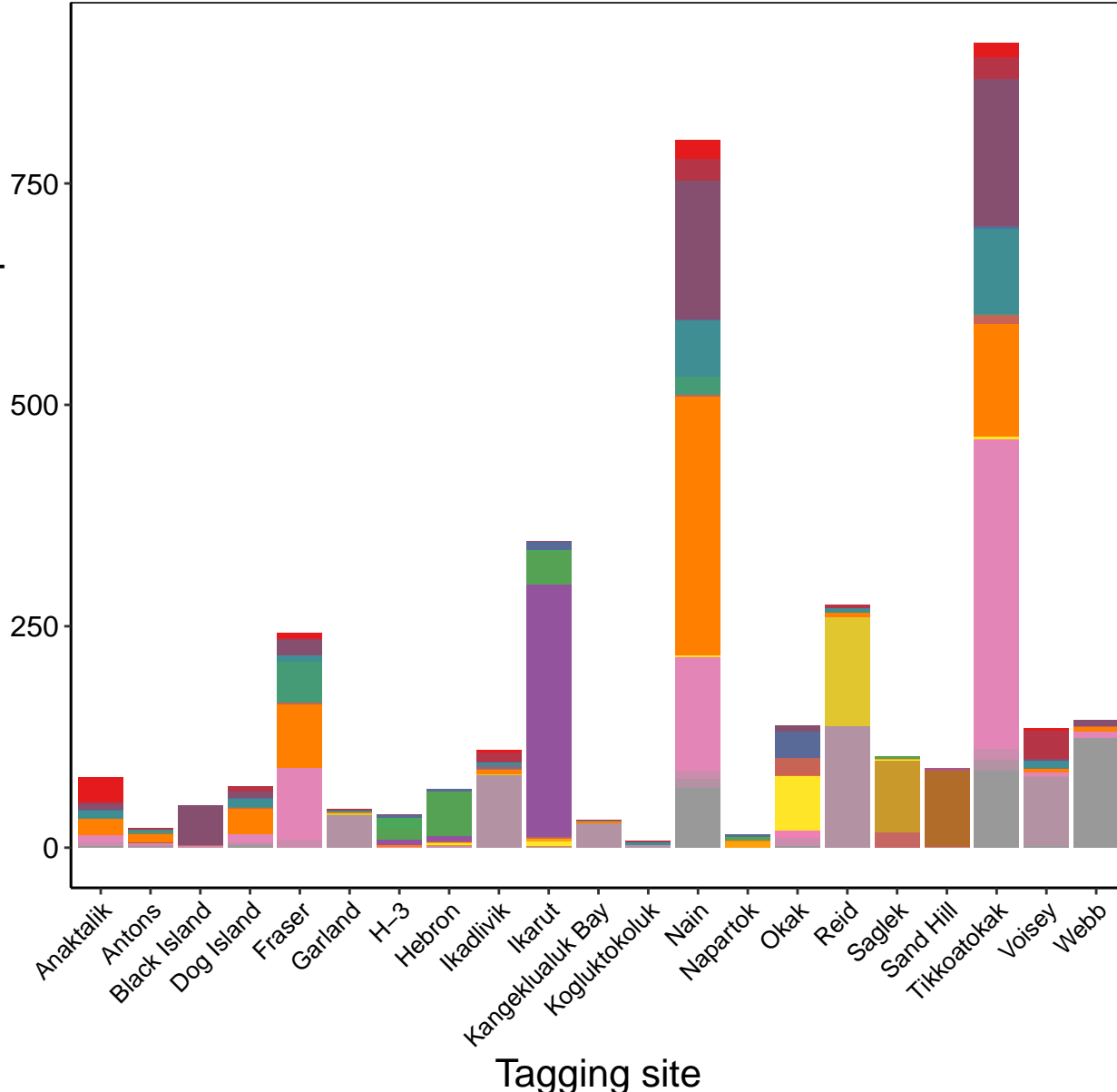

Recapture site

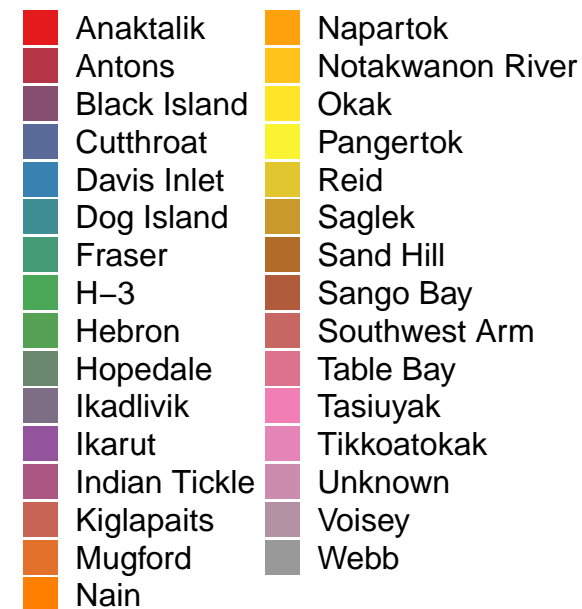

Supplement: Supplementary file 5 [file EVA-13-1055-s005.pdf]
