# Supplementary material for: Resolving fine‐scale population structure and fishery exploitation using sequenced microsatellites in a northern fish
Source: Evol Appl. 2020 Feb 20;13(5):1055–68. doi: 10.1111/eva.12922 (PMC7232759; doi:10.1111/eva.12922)
Supplement: Supplementary file 6 [file EVA-13-1055-s006.docx]

**Table S1**: Baseline and fishery samples from Newfoundland and Labrador, Canada including coordinates, population codes, sample sizes (N), and reporting units.

| **Location** | **Latitude, Longitude** | **Code** | **N**  **(µsat/SNP)** | **Drainage**  **Area (km)** | **Reporting**  **Unit** |
| --- | --- | --- | --- | --- | --- |
| *Baseline* | | | | | |
| Anaktalik Bay | 56.49753, -62.93309 | ANA | 32/30 | 1813 | ANA |
| English River | 54.96969, -59.74938 | ENG | 35/30 | 640 | ENG |
| Fraser River | 56.69082, -63.46502 | FRS | 24/23 | 1606 | FRS |
| Ikarut River | 58.16057, -63.16141 | IKA | 30/25 | 474 | IKATHRFOU |
| Ikinet River | 57.40427, -62.64107 | IKI | 28/27 | 872 | IKI |
| Ikadlivik River | 56.3126 0, -62.16876 | IKL | 17/17 | 1095 | IKLREI |
| Kamanatsuk River | 56.75377, -62.53810 | KAM | 32/29 | 829 | KAM |
| Kangalaksiorvik River | 59.38715, -64.26677 | KAN | 31/30 | 654 | KAN |
| Kingurutik River | 56.84256, -62.62164 | KIN | 27/24 | 4157 | KIN |
| Kiyuktok River | 58.39627, -62.98231 | KIY | 32/28 | 86 | KIY |
| Kogarsok River | 59.10934, -63.91097 | KOG | 32/30 | 86 | KOG |
| Komaktorvik River | 59.22694, -64.00918 | KOM | 32/29 | 699 | KOM |
| Muddy Bay Brook | 53.62977, -57.02991 | MBB | 32/26 | 344 | MBB |
| McCormick's River | 58.97814, -63.69840 | MCC | 32/30 | 311 | MCC |
| Nachvak River | 58.97935, -64.23814 | NAC | 32/30 | 680 | NAC |
| North Arm River | 58.57114, -63.49739 | NOR | 30/30 | 104 | NOR |
| Palmer River | 58.92509, -63.87748 | PAL | 30/30 | 311 | PAL |
| Pangertok River | 58.32611, -63.20870 | PAN | 32/30 | 278 | PAN |
| Parkers Bay Brook | 51.49827, -55.73268 | PBP | 14/15 | 46 | PBP |
| Puttuaalu River | 57.25526, -62.22069 | PUT | 26/24 | 1471 | PUT |
| River 103 | 58.03142, -63.03710 | R103 | 30/26 | 790 | IKATHRFOU |
| River 104 | 57.95381, -63.56003 | R104 | 30/29 | 1461 | IKATHRFOU |
| River 105 | 58.06341, -63.68450 | R105 | 31/30 | 1347 | R105 |
| River 109 | 58.22318, -63.67063 | R109 | 32/30 | 212 | R109 |
| River 78 | 55.64627, -60.68978 | R78 | 25/23 | 338 | R78 |
| Reid Brook | 56.30319, -62.08522 | REI | 10/9 | 171 | IKLREI |
| Stecker River | 58.86846, -63.45745 | STC | 30/30 | 172 | STC |
| Southwest Arm River | 58.46825, -63.64623 | SWA | 32/30 | 707 | SWA |
| *Fisheries* | | | | | |
| Black Tickle | 53.40601, -55.77281 | BTK | 3 |  | |
| Charlottetown | 52.78048, -56.10768 | CTW | 39 |  |  |
| Hopedale | 55.45720, -60.21214 | HOP | 10 |  |  |
| Lake Melville | 53.54138, -60.11650 | LMV | 14 |  |  |
| Makkovik | 55.08512, -59.17590 | MKK | 43 |  |  |
| Nain | 56.60493, -62.09372 | NAF | 637 |  |  |
| Postville | 54.88541, -59.83691 | PTV | 90 |  |  |
| Saglek Fjord | 58.52028, -63.12223 | SKF | 75 |  |  |
| St. Lewis | 52.36500, -55.79373 | SLW | 85 |  |  |
