# Supplementary material for: Resolving fine‐scale population structure and fishery exploitation using sequenced microsatellites in a northern fish
Source: Evol Appl. 2020 Feb 20;13(5):1055–68. doi: 10.1111/eva.12922 (PMC7232759; doi:10.1111/eva.12922)
Supplement: Supplementary file 7 [file EVA-13-1055-s007.docx]

**Table S2**: Rivers sampled for Atlantic salmon (*Salmo salar*) in 2016 in southern Labrador where no Arctic charr were encountered. Bold text represents rivers that are reported to have Arctic charr in Murphy and Porter (1974).

| **River** | **Latitude** | **Longitude** |
| --- | --- | --- |
| Napetipi | 51.3 | -58.05 |
| Vieux Fort | 51.32 | -58.02 |
| St Paul River | 51.47 | -57.7 |
| **Forteau River** | **51.48** | **-56.94** |
| **L'anse au Loup River** | **51.53** | **-56.82** |
| **Pinware River** | **51.63** | **-56.69** |
| **St Charles River** | **52.23** | **-55.84** |
| Port Marum | 52.4 | -55.74 |
| St. Lewis | 52.43 | -56.17 |
| **Shinnys Brook** | 52.59 | -56.34 |
| Alexis | 52.6 | -56.53 |
| Hawke River | 53.03 | -56.06 |
| Southwest Brook | 53.42 | -57.23 |
| Paradise River | 53.42 | -57.25 |
| Eagle River | 53.53 | -57.47 |
| **Sand Hill River** | **53.57** | **-56.35** |
| Big River | 54.84 | -58.94 |
| **Hunt River** | **55.57** | **-60.67** |
