# Supplementary material for: Resolving fine‐scale population structure and fishery exploitation using sequenced microsatellites in a northern fish
Source: Evol Appl. 2020 Feb 20;13(5):1055–68. doi: 10.1111/eva.12922 (PMC7232759; doi:10.1111/eva.12922)
Supplement: Supplementary file 8 [file EVA-13-1055-s008.docx]

**Table S3:** Locus name, primers, and repeat motif for microsatellite loci. Loci are named as follows: Sal(***S****alvelinus* ***al****pinus*)-MicrosatelliteType-Chromosome#-Locus#.

| **Locus Name** | **Chromosome** | **Left Oligo** | **Right Oligo** | **Repeat Motif** |
| --- | --- | --- | --- | --- |
| Sal-Tri-01-26 | 1 | ATGGCTAACTTGCGCAATGTC | ACTTTATCGACCACTGCAACG | act |
| Sal-Di-02-1535 | 2 | CTCAGTGCTAATGGGTGGAAG | CTACACGTCAGACCAGGCATG | ac |
| Sal-Tri-02-5 | 2 | AGACTACTTCAAGCACCAGAC | AGAGAAAGGGAGTGAGCTTAAC | gtt |
| Sal-Di-03-1639 | 3 | CGCCACAGGTTGCTAAGATTG | GCGTGTATTCAGAAGCAGCAC | ct |
| Sal-Di-03-1720 | 3 | GTGTGACTTTGGCCCGAG | CCGTTTAGCCAAGAAAGAGGG | ct |
| Sal-Tri-03-20 | 3 | GTGATTCACGAACGAGGGATC | TCTCACCCAGCTCTATTCTCC | agg |
| Sal-Tri-03-22 | 3 | GAGTCCCTCCAGTCCATTTATG | ATTACCACCACCGACCCTTC | agg |
| Sal-Di-05-1487 | 5 | CTTCATGCTCATCACTGGCC | CATGCACCAAACACCACTCC | ct |
| Sal-Di-05-1342 | 5 | CAAGAGGGCCAAGAGTGAGTC | AGTTAAGGAATGAAGAGGTGGG | ct |
| Sal-Tri-05-4 | 5 | GCAAATGACTTCCCTCTCTGC | CTTCTATCTCTCTCGGCCGG | cct |
| Sal-Tri-05-10 | 5 | GCACTTTGACATCAGCTGGTG | TCCAGTTCTATCACCCACCAG | agt |
| Sal-Tri-05-9 | 5 | TGCCACTGACCTTCTTCTGAC | TGTGCTTCTATGGACTTCACATG | act |
| Sal-Di-6.1-1249 | 6.1 | GGGCTGTAGTCTGGGCAG | TTTCATCCACCTGTTTCCTCC | ag |
| Sal-Di-6.1-571 | 6.1 | CCTGATGGGCACTATAGTAGC | AATAGTACGTCTGTGGGTGCC | ac |
| Sal-Tri-6.1-1 | 6.1 | CTTCGCTGCTTGAACCGC | AGCGACTTTGAGATTCTTGTTTG | aat |
| Sal-Di-6.2-542 | 6.2 | ACAACAGCAAACAAACAGTGG | CACCACACACATGCCTGC | gt |
| Sal-Di-6.2-981 | 6.2 | CATGTGCTTCAGTGTGCTCC | GCTGACCCAATTCTGAACACC | ct |
| Sal-Tri-6.2-30 | 6.2 | GCTGGGCAGATGAGACAGTAG | TGCCATGGTTTACGTCATTACC | agt |
| Sal-Di-07-251 | 7 | AGGTGAGTTGCCAGTGGAG | AGTGCTGACGTTCTCCTCAAG | ac |
| Sal-Di-07-982 | 7 | GGTATTGGTCTACTGGTGTGC | GATGACCACCAGCTTGCAC | gt |
| Sal-Tri-07-12 | 7 | CGACATGAAGGAAGCTGCAC | CTTCAGTCTGTTCAGTGGAGC | agc |
| Sal-Tri-07-1 | 7 | GCAAGACCCTCTGAAACTCTC | GGCGTGACATGCAGACTATATG | aag |
| Sal-Tri-07-14 | 7 | TTGATGAGTGTGTTGTTGCAG | CCAAATGCCTCTGTAGCGATG | aat |
| Sal-Di-08-1675 | 8 | ACACAGCTTCAAGTGTAGAGTG | TGGGTGACAAGAGACCGTG | ac |
| Sal-Di-08-1234 | 8 | ACCACACCTCAGCATGGC | AGTGTAATGTGGCTATCCTGC | ac |
| Sal-Tri-08-12 | 8 | TGGAAGTCAGTGCAAGGC | CGTATGGTTTCCGGATCCCTC | agg |
| Sal-Tri-08-3 | 8 | CCTTTGTGCTGTATTGTTTACCC | CCTGCTGTAATATCGTGGTGC | ctt |
| Sal-Di-11-1657 | 11 | AGTGGAGCAGAGAGACCATTAG | CTTAATGGTGCATCTTTCTCCTG | ct |
| Sal-Tri-11-1 | 11 | GTGGCGGCATGTACTTCTAAC | GATACAGGGTTTGACGGACTC | aag |
| Sal-Tri-11-16 | 11 | CCGTTGGCAAGGTGATTAAC | ATCAAGGTCAATGCAACGGTC | aat |
| Sal-Tri-12-2 | 12 | ATGAGAGTGCAACCCAACAAC | AACACAGACTTCCTCCCAGAC | agc |
| Sal-Di-13-1195 | 13 | GTCTGTGTGTTCTAACCGCTG | GAGGGAGACACTCTTCTTTCAG | ac |
| Sal-Di-13-189 | 13 | CTGAAGTGCGGGTGTTGC | AAACGCACTAACTGGTTAACAAG | ac |
| Sal-Tri-13-0 | 13 | ACAAACATAACCCAGGACCAC | AGATCTGGCACACTTCTCCTG | acc |
| Sal-Tri-13-16 | 13 | TGGTGGATGACAATGACAATGAG | AGCTTACCTCCTCATCAGTGG | agg |
| Sal-Tri-13-5 | 13 | CGTCATCATACTCACCGTCG | TGTAGCCAATGAAAGACAGCG | atc |
| Sal-Tri-14-22 | 14 | TGCCAACACCTACAGAGAAC | TCTGTAGTTGTTGGCAGCAG | act |
| Sal-Tri-14-6 | 14 | CTAATGTGGCTGAGTCAATTGC | TCGTCGTTCATGTCCTTATCATC | gat |
| Sal-Tri-14-1 | 14 | ATGAGAGACCTGCACCTCCC | AGCTCACAACAGTAGGGAGAG | ctt |
| Sal-Tri-14-3 | 14 | ACAGTCGAGGATTCAGGACAG | CTTTGCCGATCTCTTCAGCTG | gat |
| Sal-Di-15-51 | 15 | TCTTGTGATTCTGGACGTGTG | CAGATGTAGCCTCACACCAAC | gt |
| Sal-Di-15-102 | 15 | GAGTAGCGTTCGTACTCACAC | TGGTAAGCTCTCTCCAGTGTTC | ac |
| Sal-Di-16-475 | 16 | AGAGTGGAGGCTGTGTATGC | TCACAAACACGCTCGCTTG | gt |
| Sal-Tri-16-9 | 16 | CCTGCAGCAGTAAGAGAGGAC | GTAGATCATGGCCCAGGGTG | cct |
| Sal-Tri-16-13 | 16 | AGTTGTGAATGCTTAACTACAGC | CCAGTAGAAGTTATAGTAGGCCC | aat |
| Sal-Tri-16-8 | 16 | CCCATTTATCTGCCTCTTTCATG | GCTGTAGTCATGGCCTTATCTG | gat |
| Sal-Di-17-441 | 17 | TGTCTTCAGGCAAAGGAGTC | GTCATGTTGGCCTCCTTCTG | gt |
| Sal-Tri-17-17 | 17 | GGTTCTCCAGGGTCTGCAG | GAGGAGTACGCCCACACAG | cct |
| Sal-Tri-17-24 | 17 | GGTCTTCCTGTTTCCTGTTTCC | CCAAAGTGAACATTTCCAGTGTG | att |
| Sal-Tri-17-3 | 17 | GCCTTGACCACTGCCATG | ACTGCGAACGTCCTCTGG | acc |
| Sal-Tri-18-13 | 18 | ATCCCAGCGGTTGTTGAC | CATCTGTAGCCGCCGATTAC | gct |
| Sal-Di-19-1255 | 19 | TCTCTGTCTGTCCGTCCG | TCGCAAAGTGACATAACCCTTC | ct |
| Sal-Tri-19-4 | 19 | AGGTAGGTAGACAGACACATAGC | TGGCCTGTCTCTGTAGTACTG | atc |
| Sal-Tri-19-22 | 19 | TGCTGGCATAACGTTAGCTAG | ACATTTGTGTTTGCTTGCCAG | act |
| Sal-Tri-19-5 | 19 | ACCAATGTCACCAATACCACG | CTCGTCAAAGGATGTGGTCAC | atc |
| Sal-Di-20-1207 | 20 | CCTTCACTATGGCTGGGCAC | GGAGCGACATATTTCAACTCAAG | ac |
| Sal-Di-20-1836 | 20 | ACACATGGTCAGGAAGGAAATG | GGCATACAGAGCAGAGTGAATC | ac |
| Sal-Tri-20-21 | 20 | GAGAGCGAGGGATTGGTATGG | GACAACCAGCCACCTCCC | agg |
| Sal-Tri-20-30 | 20 | CTGGACCGGCATAATACAGAAG | TTCACGTTTGTTCCTTTCCCG | att |
| Sal-Tri-20-5 | 20 | TGGCCACATATGCTCCCG | CTTGTGATCGCCAACCACC | ctt |
| Sal-Tri-20-12 | 20 | AAGCCTCTGTAACTGCAAGTG | CATTAATTTCAGCTCCTTGTGGC | gat |
| Sal-Tri-20-19 | 20 | CCTCCACCTCTTCTTTCACAC | CCTCATCCTGGAACGAGTACC | cct |
| Sal-Di-22-151 | 22 | AATTTCCACACAACCGAGCTG | CAGACGAACAGACACAAGCG | gt |
| Sal-Tri-22-20 | 22 | CCATCATTGAGAGCTAGCTGTG | GAGCTCCCAAGTCTAGTGTTTC | att |
| Sal-Tri-22-14 | 22 | CAGCTAAATGACACCCTCTCC | CCCAGATCTAGCAGGATGAATC | cct |
| Sal-Tri-22-2 | 22 | ACAGTGAAGCCTCTAACCTCG | GTGTTACATAGAAAGTTGCTCCC | aag |
| Sal-Tri-22-7 | 22 | TTGATGACCAGAAACAGCGTC | AACTAGGAGAGCACCCTTGAC | atc |
| Sal-Tri-23-0 | 23 | GCCGATAAACACCACCGAAG | TCCTGGCTTGATTGAATACTTGC | aag |
| Sal-Tri-23-5 | 23 | GCGCTTCTGTTCTATCCTCAC | AGCTCGACTTGCAGGACTC | atc |
| Sal-Tri-24-1 | 24 | GTGACCGGCTTGTTCCATTC | TGCCTCGTACTCACTACAGAC | aag |
| Sal-Di-25-11 | 25 | TATCAAGAGACCTGCTGTGTG | GGTTAGCTGAGAAGTGTGTGG | ac |
| Sal-Di-25-336 | 25 | TTGATACGGTATGCGCATTTG | AGTGGCAGCTGGTTGGATG | gt |
| Sal-Tri-25-5 | 25 | GGAAGGCCAGTACAATATTGAGG | TTGGTTACTGCGGTTATGGTC | gat |
| Sal-Di-26-1493 | 26 | GTTATGTCCCTCCCAGCTGTC | GGGACTGAGGCGAGTCAAC | gt |
| Sal-Di-26-780 | 26 | GTGCCGATATTAGAGACCCAAG | CTCGATTAGCATTGCACCGG | ac |
| Sal-Tri-26-3 | 26 | TTTCTGGATAACACTTGGCGG | TGAGAACCACTGGCTCATTAG | acc |
| Sal-Tri-26-14 | 26 | TGGCTGTGCTGTGAATGATTC | TCACCACCACCTCGCTATG | agc |
| Sal-Di-27-214 | 27 | TTCACGACGCGACCTGAG | CGCTACTGTGTGGTTTGGATC | gt |
| Sal-Di-27-39 | 27 | GTGCATCATTCACAGCCATATG | CCCAACATGACAGGCAGG | ac |
| Sal-Tri-27-8 | 27 | CCATTTCCATTTCACACCTCAC | AGGTGTGGTTGATGTAGGCC | aac |
| Sal-Di-28-1221 | 28 | TGACAACACATCAGGCGG | GTGCTGTATATTCACTGGGATTG | ct |
| Sal-Tri-28-0 | 28 | CTCGGTGACTTTCCCAGATTC | TCCAGAGTCTACAACAATGACC | ctt |
| Sal-Tri-28-9 | 28 | CGGCCAATAGTCCTGTTCATC | ACTTTCCTAAACAAGCACCATTG | att |
| Sal-Di-30-1164 | 30 | GCTTGATTCGCTGTTGTCTC | AAATCCATGTTTACAGTCACGC | ct |
| Sal-Di-30-844 | 30 | GCAGGAGAAAGGGAACACAAG | CTGGTCTTCACGTGTGTATGC | gt |
| Sal-Tri-30-1 | 30 | GATGACACTGCAAACTGGGAG | AAAGCTCCAAGTGCCTCCTTC | aag |
| Sal-Tri-30-10 | 30 | TCCTTAGTGTTCCGAAAGAGGG | TCCTTCGCCTTGCTTGTTAAC | agg |
| Sal-Di-31-422 | 31 | CACCATTAGCCAGCGGTG | ACCTTGTTCTGCTCCATCAC | gt |
| Sal-Tri-31-9 | 31 | AAAGCGCTATATAGGTCCCATG | CACTCCAAGCTGACAATCATATG | att |
| Sal-Tri-31-1 | 31 | TTCCATCTCTACAGCTGGTCC | AACCATTTCCCGTTTCATATGC | atc |
| Sal-Di-32-188 | 32 | AGGAGTTTGTCTGGGCACAC | GACAGACAGTAACCATCCATAGC | ac |
| Sal-Di-32-884 | 32 | CTCTGTCTGTAGAACATGGTGG | GGGTGAGTGAAAGCGTATGAC | ac |
| Sal-Di-33-1038 | 33 | TCGGTTTCAGTTGACTCCCAC | CCTGCCTCCTGTTCTTTCAAG | ac |
| Sal-Tri-33-13 | 33 | TGGAACTCAGACATGTTACCAG | GCAGGGCTTTGGAGAGGG | gct |
| Sal-Di-35-679 | 35 | CCTCAACTGCACGGAATCATC | GAAATGGCCAGTCACTCAGAG | ac |
| Sal-Di-36-244 | 36 | GCTGTCTGTCACACCTCAAAC | GGCGTGTTAGGTCTATTTCTCC | ac |
| Sal-Di-36-765 | 36 | AGGAATATCATCACAGTGCAGC | AGTCTGTCATTTCCTGCTCTTG | ac |
| Sal-Di-37-588 | 37 | CATAAATAATAGGGCTGCACACG | AACACACAGAGATGGGTTTGC | ac |
| Sal-Di-4p-1163 | 4p | AAATGCAAATGAGCCTGGTGG | GAGAGGGTGAGGTCGGAG | ct |
| Sal-Di-4p-165 | 4p | CTTCTTCAAAGCCAGCCACTC | GACTAAGACAGGCCAGGGAG | gt |
| Sal-Di-4p-585 | 4p | TTGTTCGTTGCCTATTCTCCC | GTGGTCAAGGTCCGTTGATG | gt |
| Sal-Tri-4p-11 | 4p | CAGGACAACTATAAGCACCCAC | CTCACTTTCACCTGTAGAATTGC | att |
| Sal-Tri-4p-14 | 4p | ACGTGGTGTATGAACTGACAC | GCTGAGATGGTTGTATGTCCC | aat |
| Sal-Di-4q-352 | 4q | GAGTTATTGCGTGTGTGTCCC | CACTGTGTCCCTTTGTAACCC | gt |
| Sal-Tri-4q-11 | 4q | ACCACGGTGATTGTTTCCTTG | ACCTCACCGTTTACTAGAAGAGG | cct |
| Sal-Tri-4q-22 | 4q | CCCAAGATCCTGGATGCTC | GATTTGTCCTGAGCTGCTGAG | agc |
| Sal-Tri-4q-32 | 4q | AGCCTGCACTTTCTATCACTG | TACAAAGTGACATCTGTGCCC | aat |
| Sal-Tri-4q-7 | 4q | AGCTGTGAGGGAATGTTGTTG | CCCTCCTTCTCAGTTCTCATATC | gat |
| Sal-Tri-4q-9 | 4q | GGACAAGCTACATTGGTGGTG | CTCCAGATCCACAGCCACTAC | gat |
| Sal-Tri-4q2-12 | 4q2 | AGAACAGGTATAAGCAACCACC | CAGAATCACTCATAAGGACCAGG | att |
| Sal-Tri-4q2-2 | 4q2 | CCGCATCTCACCACAGACTC | TCCATGACGTTCTATGTGTCTG | atc |
