# Supplementary material for: Resolving fine‐scale population structure and fishery exploitation using sequenced microsatellites in a northern fish
Source: Evol Appl. 2020 Feb 20;13(5):1055–68. doi: 10.1111/eva.12922 (PMC7232759; doi:10.1111/eva.12922)
Supplement: Supplementary file 9 [file EVA-13-1055-s009.docx]

**Table S4**: Pairwise *F*_ST_ (WC84) values estimated from microsatellites (lower matrix) and SNPs (upper matrix) in the *hierfstat* package (Goudet, 2005) in R.
