# Supplementary material for: Resolving fine‐scale population structure and fishery exploitation using sequenced microsatellites in a northern fish
Source: Evol Appl. 2020 Feb 20;13(5):1055–68. doi: 10.1111/eva.12922 (PMC7232759; doi:10.1111/eva.12922)
Supplement: Supplementary file 10 [file EVA-13-1055-s010.docx]

**Table S5**: Tag and recapture data for Arctic charr, including the number of individuals tagged and recaptured, and the percent recaptured from their original tagging site for each of 21 sites.

| **Region** | **Tag Site** | **N Tagged** | **N Recaptured** | **% Recaptured from tag site** |
| --- | --- | --- | --- | --- |
| Hebron | H-3 | 420 | 38 | 28.9 |
| Hebron | Hebron | 411 | 66 | 75.8 |
| Hebron | Ikarut River | 2498 | 346 | 82.1 |
| Nain inshore | Anaktalik Bay | 290 | 79 | 34.2 |
| Nain inshore | Fraser River | 807 | 243 | 18.9 |
| Nain inshore | Nain Bay | 3085 | 799 | 36.5 |
| Nain inshore | Tikkoatokak Bay | 2257 | 909 | 38.2 |
| Nain inshore | Webb Bay | 312 | 144 | 86.1 |
| Nain offshore | Black Island | 285 | 48 | 93.8 |
| Nain offshore | Dog Island | 345 | 69 | 13.4 |
| Napartok | Napartok | 228 | 15 | 46.7 |
| Okak | Okak | 505 | 138 | 44.2 |
| Saglek | Saglek | 1317 | 103 | 77.7 |
| Sand Hill | Sand Hill River | 207 | 90 | 95.6 |
| Voisey | Antons | 216 | 22 | 11.1 |
| Voisey | Garland Bight | 158 | 44 | 0 |
| Voisey | Ikadlivik | 411 | 110 | 3.6 |
| Voisey | Kangeklualuk Bay | 139 | 31 | 0 |
| Voisey | Kogluktokoluk Bk | 127 | 8 | 0 |
| Voisey | Reid Brook | 1333 | 274 | 44.5 |
| Voisey | Voisey Bay | 599 | 135 | 58.2 |
